# Metastatic Cancers Exploit Vagal Sensory Neurons and Neural Repair Programs to Colonize New Tissues

**DOI:** 10.21203/rs.3.rs-10437078/v1

**Published:** 2026-07-27

**Authors:** Tiago H Zaninelli, Telma Saraiva-Santos, Gustavo Gastã0 Davanzo, Alliny CS Bastos, Ruben Silva, Jose C de Lima-Junior, Amanda Martins Dionisio, Soraia Mendes-Pierotti, Kennedy K Reed, Yuriy Kirichok, Rubia Casagrande, Waldiceu A Verri, Igor Smirnov, Jason Weber, David Y Chen, Jonathan Kipnis, Felipe A Pinho-Ribeiro

**Affiliations:** 1 Department of Medicine, School of Medicine, Washington University in St. Louis, Saint Louis, MO, USA; 2 Brain Immunology and Glia (BIG) Center, Washington University in St. Louis, Saint Louis, MO, USA; 3 Department of Pathology and Immunology, School of Medicine, Washington University in St. Louis, Saint Louis, MO, USA; 4 Division of Medical Oncology, Washington University in St. Louis, Saint Louis, MO, USA; 5 Department of Biochemistry & Molecular Biophysics, Washington University in St. Louis, St. Louis, MO, USA; 6 Department of Pharmaceutical Sciences, Center of Health Sciences, Londrina State University, Londrina, Paran·, Brazil; 7 Department of Immunology, Parasitology, and Pathology, Center of Biological Sciences, Londrina State University, Londrina, Paran·, Brazil

## Abstract

Metastasis remains the leading cause of cancer-related mortality, yet the mechanisms that enable disseminated tumor cells to colonize distant organs remain incompletely understood.^1,2^ Here, we identify vagal sensory neurons as key facilitators of metastatic outgrowth. Using genetic, pharmacologic, surgical, and tissue-targeted denervation strategies in murine models of metastatic melanoma and breast cancer, we show that depletion of vagal sensory inputs markedly reduces pulmonary colonization. Mechanistically, metastatic cells exploit nerve injury-induced protein 1, NINJ1, to engage vagal afferents and activate a β-catenin-dependent transcriptional program. This interaction increases expression of receptors for growth factors and enables cancer cells to respond to macrophage- and fibroblast-derived trophic signals within the metastatic niche. Unbiased co-immunoprecipitation proteomics revealed that NINJ1 sequesters key components of the β-catenin destruction complex, including DVL1, AXIN1, CK1δ/ε, and CK2. Consequently, the presence of vagal sensory neurons leads to an NINJ1-dependent accumulation of active β-catenin form in cancer cells, while pharmacologic inhibition of β-catenin signaling abolished NINJ1-driven growth factor responsiveness. Together, these findings identify a neuro-metastatic axis in which metastatic cells co-opt a vagal sensory neuron-associated repair program to colonize new tissues. Targeting NINJ1 significantly reduced metastatic outgrowth across multiple cancer models and host backgrounds, indicating a broad therapeutic potential of these findings.

## INTRODUCTION

Cancer metastasis is the primary cause of cancer-related deaths worldwide, accounting for over 90% of fatalities^1,2^. This lethality is mainly driven not by the primary tumor, but by the subset of cancer cells that successfully colonize distant organs, compromising their vital functions ^3^. Metastasis is a multistep biological process in which cancer cells escape the primary tumor site, invade surrounding tissue, enter and survive within the circulation (bloodstream or lymphatics) or along the nerves (perineural invasion), extravasate at distant sites, and ultimately colonize the new tissue, disrupting tissue homeostasis and the function of vital organs ^4^. A critical challenge in modern oncology is that many disseminated cancer cells evolve highly effective immune-evasive properties, enabling them to escape detection and destruction by the host's immune system ^5-7^. Consequently, even promising treatments like immunotherapy are often ineffective in patients with advanced metastatic disease, a clinical reality that underscores the urgent need to identify and target the additional mechanisms that enable these evasive cells to survive and proliferate in new tissues^8^.

The journey from a primary tumor to a macroscopic metastasis is a remarkably inefficient process, with the most significant barrier occurring after cancer cells have extravasated into a distant organ ^9^. While genetic and epigenetic alterations within tumor cells contribute to their immune-evasive properties and metastatic potential^10,11^, successful colonization of distant sites requires dynamic adaptation to organ-specific cues ^12,13^. This 'post-extravasation bottleneck' is the primary rate-limiting step of the metastatic cascade, characterized by an attrition rate that can exceed 99% ^14^. This inefficiency is not due to failures in circulation or extravasation, but rather to the profound challenge individual cancer cells face in surviving and initiating proliferation within a foreign tissue microenvironment. The vast majority of immune-evasive disseminated tumor cells either perish shortly after arrival or enter a state of long-term dormancy ^15,16^. Therefore, the formation of a lethal metastasis is a rare event, accomplished only by the small fraction of cancer cells that can successfully co-opt local survival cues to overcome this bottleneck. Understanding the mechanisms that empower these rare metastatic cells is paramount to preventing metastasis-related deaths.

Certain cancers exhibit a remarkable ability to interact with sensory nerves, a phenomenon that was pivotal to some of the earliest foundational discoveries in modern neuroscience. In 1954, Rita Levi-Montalcini and Stanley Cohen identified nerve growth factor (NGF) as the neurotrophic molecule produced by cancer cells capable of inducing robust axonal outgrowth, a finding that emerged from experiments using mouse sarcoma tumors^17^. Patients with tumors exhibiting nerve infiltration or perineural invasion face a higher risk of metastatic disease and recurrence ^18-22^, suggesting that cancers that co-opt neuronal signals gain a remarkable ability to proliferate in new organ sites. However, the underlying mechanisms behind this correlation remain poorly understood. Sensory neurons, traditionally associated with transmitting sensory information, play major roles in tissue homeostasis and repair ^22,23^. After injury, sensory neurons contribute to tissue repair by regulating immune cell activity ^23-25^, supporting neovascularization ^25,26^, and maintaining the stem cell niche ^27,28^. These properties make sensory neurons potentially attractive targets for infectious pathogens and cancer cells ^21^. In prior work, we discovered that bacterial pathogens can exploit sensory neurons to modulate macrophage and neutrophil function and facilitate their ability to colonize host tissues ^29,30^. While the recent advances in cancer neuroscience started to elucidate the impact of sensory neurons in tumorigenesis and cancer immune evasion, their influence in the clinical scenario of metastatic tumors that have already escaped immune recognition and are resistant to therapy remains largely unexplored.

Herein, we aimed to address this critical knowledge gap and mechanistically dissect the interplay between host sensory neurons and immune-evasive tumor cells in the post-extravasation bottleneck. Vagal sensory afferents densely innervate the lung at multiple anatomical levels, including the airway epithelium, smooth muscle, and vasculature, where they coordinate reflexes such as coughing and bronchospasm ^31-33^. The discovery of the vagal inflammatory reflex by Tracey and colleagues^34^ established the vagus nerve as a central regulatory hub controlling both immune and non-immune components of visceral tissues, fundamentally reshaping our understanding of neuroimmune communication. A series of very sophisticated studies subsequently demonstrated that vagal and nociceptive sensory neurons shape pulmonary immune responses and tissue homeostasis during infection, inflammation, and injury, including bacterial pneumonia, influenza, and fibrotic disease. ^35-40^ Despite these advances, whether and how vagal sensory neurons contribute to the survival and outgrowth of immune-evasive metastatic cancer cells in secondary tissues remains largely unexplored. We identified vagal sensory neurons as important tissue facilitators of metastatic colonization in the lungs. The lungs represent a frequent and often fatal site of metastasis for multiple cancers, including melanoma and breast cancer^41^. Like other viscera commonly affected by metastatic disease, lung tissue is densely innervated by sensory neurons that originate from the vagal ganglia (nodose and jugular ganglia) ^42^. These interoceptive neurons play critical roles in tissue homeostasis and repair following injury, ^35,40,43,44^ which led us to investigate their possible influence on lung metastasis. Using genetic and pharmacological denervation strategies in different murine models of metastatic disease, we demonstrate that ablation of vagal sensory inputs robustly impairs metastatic outgrowth in the lungs. We further uncover that tumor cells engage a tissue repair program initiated by neurons via nerve injury–induced protein 1 (NINJ1), a cohesion molecule enriched in both cell types (cancer cells and vagal sensory neurons). This NINJ1-mediated interaction serves as a critical 'handshake' between metastases and vagal afferents, promoting a stem-like transcriptional program in cancer cells and enhancing their responsiveness to growth factors produced by tissue resident cells within the lung microenvironment. We further use a combination of immunoprecipitation, proteomics, and transcriptomics to provide an unbiased characterization of NINJ1’s novel signaling function and uncover that its effects on cancer cells are mediated by the activation of β-catenin. Targeting NINJ1 impairs the ability of metastatic cells to survive and proliferate in the new tissue, significantly reducing metastatic outgrowth. Together, our findings define a novel neuro-metastatic axis that is critical for overcoming the post-extravasation bottleneck and can be therapeutically targeted to disrupt tumor progression in organs innervated by vagal sensory neurons.

## RESULTS

### Vagal Sensory Neurons Promote Lung Metastasis

**Extended Data Figure 1.**
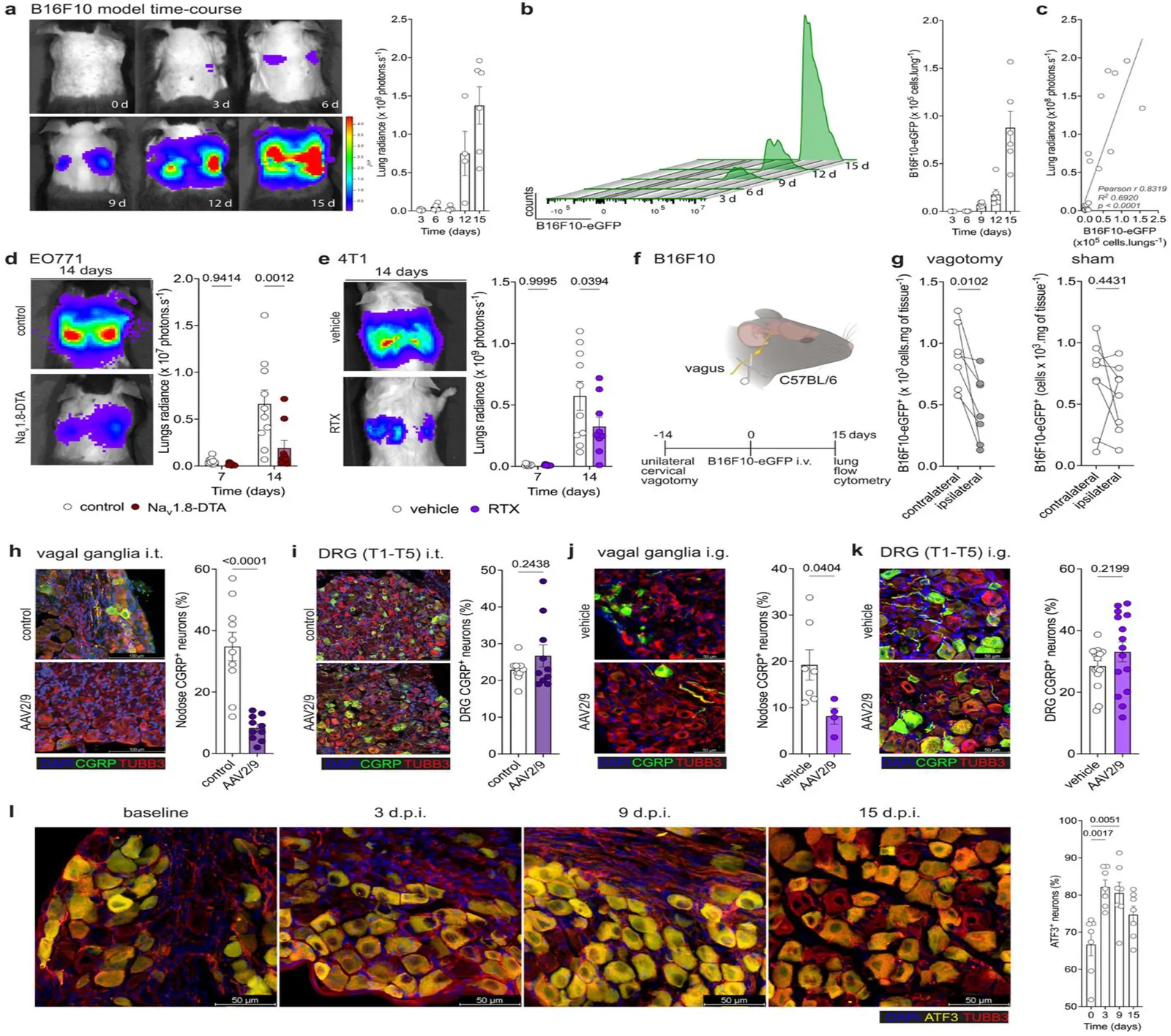
Additional characterization of sensory neurons in cancer lung metastasis. **a.** Time-course bioluminescence imaging (BLI) images and quantification of lung radiance in mice with melanoma lung metastasis. n = 4–6 mice per group. **b.** Representative flow cytometry histogram and quantification of melanoma cells in lung tissue over time. n = 4–6 mice per group. **c.** Pearson correlation between lung BLI radiance and the number of B16F10-eGFP tumor cells 15 days post-injection. n = 4-6 mice per group. **d.** Representative BLI images and radiance quantification of EO771 breast cancer lung metastasis in control and Nav1.8-DTA mice. n = 10 mice per group. **e.** Representative BLI images and radiance quantification of 4T1 breast cancer lung metastasis in vehicle- and resiniferatoxin (RTX)-treated mice. n = 10 mice per group. **f.** Schematic representation of the experimental design for unilateral vagotomy. **g.** Quantification of B16F10-eGFP cells in the lungs of unilateral vagotomy and sham mice. **h.** Validation of TRPV1^+^ sensory neuron depletion: representative immunohistochemistry (IHC) images of vagal ganglia and quantification of the percentage of CGRP^+^ neurons in animals receiving intratracheal (i.t.) instillation of vehicle (empty vector) or AAV2/9-TRPV1-DTA. n = 10 mice per group. **i.** Validation of TRPV1^+^ sensory neuron depletion: representative IHC images of thoracic dorsal root ganglia (DRG) and quantification of the percentage of CGRP^+^ neurons in animals receiving intratracheal (i.t.) instillation of vehicle (empty vector) or AAV2/9-TRPV1-DTA. n = 10 mice per group. **j.** Validation of TRPV1^+^ sensory neuron depletion: representative IHC images from vagal ganglia stained for CGRP and quantification of the percentage of CGRP^+^ neurons from intraganglionic (i.g.) vehicle (empty vector)- and AAV2/9-injected animals. n = 4-7 mice per group. **k.** Validation of TRPV1^+^ sensory neuron depletion: representative IHC images from thoracic dorsal root ganglia (DRG) stained for CGRP and quantification of the percentage of CGRP^+^ neurons from intraganglionic (i.g.) vehicle (empty vector)- and AAV2/9-injected animals. n = 4-7 mice per group. **l.** Time-course IHC images from vagal ganglia stained for ATF3 and βIII tubulin (TUBB3) and quantification of the percentage of ATF3^+^ neurons. n = 6-7 mice per group. Data are shown as mean ± SEM. Statistical significance was determined by Pearson correlation coefficient (c), two-way ANOVA (d and e), paired two-tailed Student’s t-test (g), unpaired two-tailed Student’s t-test (h, i, j, and k), and one-way ANOVA (l). RTX, resiniferatoxin; i.v., intravenous injection; i.t., intratracheal; i.g., intraganglionic.

**Figure 1.**
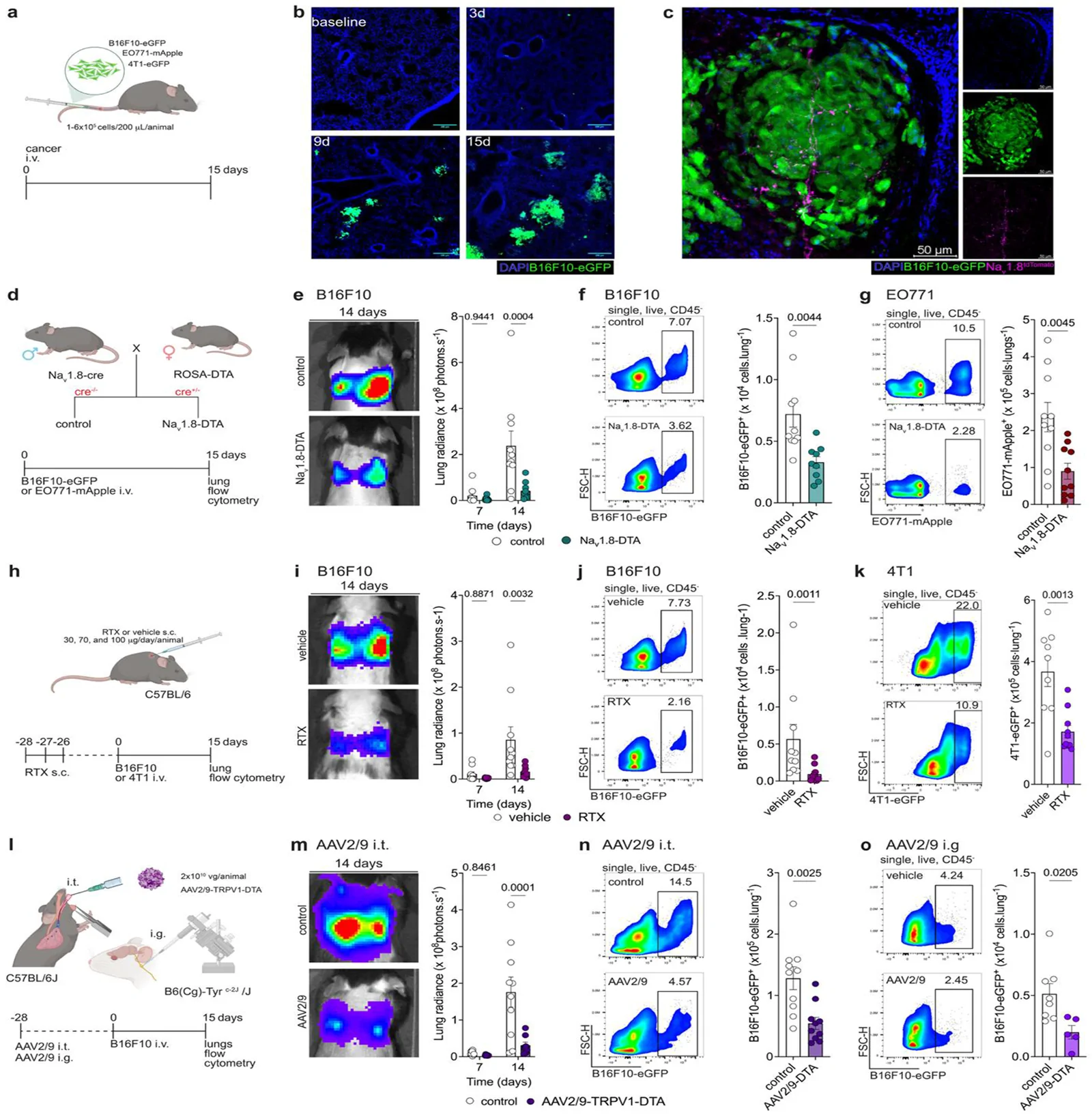
Vagal sensory neuron innervation promotes melanoma cancer lung metastasis. **a.** Experimental design of the hematogenous mouse model of tumor metastasis. **b.** Representative images of time-course lung immunohistochemistry showing B16F10-eGFP metastatic tumor outgrowth in green. **c.** Higher-magnification representative image showing a cluster of metastatic melanoma cells (GFP+) near sensory fibers (Nav1.8+) in lung tissue. **d.** Experimental design and timeline for Nav1.8 sensory neuron depletion. **e.** Representative bioluminescence imaging (BLI) images and radiance quantification. n = 10 mice per group. **f.** Representative flow cytometry panels and quantification of tumor cells in the lung tissue from control and Nav1.8-DTA mice. n = 10 mice per group. **g.** Representative flow cytometry panels and quantification of EO771 tumor burden from control and Nav1.8-DTA mice. n = 10 mice per group. **h.** Experimental design and timeline for pharmacological depletion of TRPV1+ sensory neurons using RTX. **i.** Representative BLI images and radiance quantification. n = 10 mice per group. **j.** Representative flow cytometry panels and quantification of tumor cells in the lung tissue from vehicle- and RTX-treated mice. n = 10 mice per group. **k.** Representative flow cytometry panels and quantification of 4T1 metastasis in vehicle- and RTX-treated mice. n = 9–10 mice per group. **l.** Experimental design and model timeline for intratracheal instillation (i.t.) and intraganglial (i.g.) injection of AAV2/9-TRPV1-DTA. **m.** Representative BLI images and lung radiance quantification following AAV2/9-TRPV1-DTA treatment. n = 6–10 mice per group. **n.** Representative flow cytometry panels and quantification of tumor cells in lung tissue. n = 6–10 mice per group. **o.** Representative flow cytometry panels and quantification of tumor cells in the lung tissue of vehicle- and AAV2/9-TRPV1-DTA-injected mice. n = 4-8 mice per group. Data are shown as mean ± SEM. Statistical significance was determined by two-way ANOVA (e, i, and m); unpaired two-tailed Student’s t-test (f, g, k, n, and o); and Mann–Whitney test in (j). i.v., intravenous injection; IHC, immunohistochemistry; DTA, diphtheria toxin A; RTX, resiniferatoxin; s.c., subcutaneous injection; vg, viral genomes.;

Metastatic cancer cells frequently acquire highly immune-evasive properties that allow them to disseminate without efficient immune clearance and limit the efficacy of therapies^5,6,11^. This feature, however, does not explain the ability of a very small number of cells to colonize new tissues. Identifying local factors that support the survival and proliferation of these immune-evasive cells in new tissues is therefore crucial to developing therapies for patients facing this advanced stage of metastatic disease. To specifically investigate the role of sensory neurons in this process, we employed well-established lung colonization models using three aggressive, poorly immunogenic, murine metastatic cancer cell lines – B16F10 melanoma, and EO771 and 4T1 breast carcinomas – injected intravenously in their respective syngeneic, immunocompetent hosts. This approach provides a clinically relevant context in which tumor, stroma, and a functional immune system are genetically matched, allowing us to isolate the host factors that support the survival and proliferation of cancer cells that have already bypassed immune surveillance and disseminated. We first established a model of melanoma lung colonization by injecting intravenously 6 x 10^5^ B16F10 cells stably expressing eGFP and luciferase (B16F10-GFP-Luc) (Fig. 1a). B16F10 is a highly metastatic derivative of the B16 melanoma line originally isolated from a spontaneous melanoma in C57BL/6 mice^45^. The B16F10 variant was selected for its high metastatic potential, particularly to the lungs, through repeated *in vivo* passages in mice ^45^. After tumor injection, luciferase signal progressively increased in the lungs over time (Extended Data Fig. 1a) and correlated closely with the number of GFP+ tumor cells quantified in lung tissue by flow cytometry during a 15-day time course (Extended Data Fig. 1b-c). Day 15 was used as the humane endpoint because mice developed severe illness beyond this point. Immunohistochemical analysis of lung sections revealed a temporal pattern in which isolated tumor cells observed at early time points (day 3) were progressively replaced by multicellular clusters (at days 9 and 15) (Fig. 1b), consistent with local metastatic outgrowth after seeding. We identified metastatic foci located adjacent to sensory fiber bundles and infiltrated by small individual fibers (Fig. 1c**, Supplementary Video 1**), raising the possibility that direct neuron-cancer interactions may occur (Fig. 1b). To test whether sensory neurons influence metastatic outgrowth, we used complementary genetic and pharmacologic approaches targeting the sensory neuron markers *Scn10a* (Nav1.8) and *Trpv1*, both of which are expressed by lung-innervating sensory neurons. ^22,46 29,30 47^ Nav1.8 is a voltagegated sodium channel predominantly expressed in peripheral sensory neurons ^22,46^. Genetic ablation of Nav1.8+ neurons was achieved by crossing Nav1.8-Cre mice (B6.129(Cg)-Scn10a^tm2(cre)Jwo/TjpJ^) with Rosa-DTA mice (B6.129P2-Gt(ROSA)26Sor^tm1(DTA)Lky/J^) to generate Nav1.8-cre^+/−^ DTA^+/−^ animals with systemic loss of Nav1.8+ sensory neurons, alongside littermate controls (Fig. 1d). Sensory neuron depletion markedly reduced B16F10 metastatic burden in the lung, as measured by both bioluminescence imaging and flow cytometry (Fig. 1e-f), indicating that metastatic outgrowth is facilitated by the presence of sensory neurons. To establish that this effect was not confined to a single tumor type, we extended the analysis to another model of metastatic cancer induced by EO771 cells. The EO771 cell line is a murine mammary cancer model derived from a spontaneous tumor in C57BL/6 mice ^48^. It is frequently used in breast cancer research due to its ability to mimic human breast cancer subtypes, particularly the luminal B subtype, which is associated with a poor prognosis ^48^. Genetic depletion of Nav1.8+ sensory neurons also significantly reduced EO771 cancer lung metastasis (Fig. 1g, Extended Data Fig. 1d). We next used a complementary post-developmental denervation strategy targeting TRPV1+ neurons. Transient receptor potential vanilloid subtype 1 (TRPV1) is a non-selective calcium ion-dependent cation channel known as the capsaicin receptor (the active ingredient of pepper spray) and is also expressed by sensory neurons that innervate the lungs ^47^. Pharmacological depletion of TRPV1+ neurons was achieved by treating 1-month-old mice with escalating doses (30, 70, 100 μg/kg) of resiniferatoxin (RTX), a high-affinity toxin that binds to TRPV1 and leads to post-developmental denervation and eventual loss of sensory neurons, ^29,30^ beginning four weeks before tumor inoculation (Fig. 1h). Compared with vehicle-treated controls, RTX-treated mice developed significantly fewer B16F10 lung metastases (Fig. 1i-j). To ensure our findings were not specific to the C57BL/6 genetic background, we replicated these experiments using the 4T1 mammary carcinoma model in BALB/c mice. The 4T1 cell line is a highly aggressive, triple-negative mammary carcinoma derived from BALB/c mice and that, like EO771, is widely used to mimic stage IV human breast cancer because of its ability to rapidly disseminate and establish metastasis in immunocompetent syngeneic hosts ^49^. Depletion of sensory neurons in BALB/c mice with RTX also significantly impaired lung metastatic outgrowth in the 4T1 breast cancer model (Fig. 1k, Extended Data Fig. 1e). Collectively, these findings demonstrate that depletion of sensory neurons compromises pulmonary metastatic outgrowth across multiple host strains (C57BL/6; BALB/c) and tumor types (B16F10 melanoma; EO771/4T1 breast), highlighting the broad significance of sensory neurons in lung colonization by metastatic cancer cells.

Because the lungs are primarily innervated by vagal sensory afferents arising from the jugular-nodose complex ^31-33^, we next asked whether this population accounts for the phenotype observed in the systemic depletion models. Unilateral cervical vagotomy significantly reduced the number of tumor cells in the denervated ipsilateral lung relative to the contralateral side in the same animal (Extended Data Fig. 1f,g). To distinguish vagal afferents (interoceptive) from thoracic dorsal root ganglion (DRG) neurons (somatosensory), which also express Nav1.8 and TRPV1, we next used two complementary, Adeno-Associated Virus (AAV)-based strategies to deliver a TRPV1 promoter-driven diphtheria toxin subunit A (DTA) expression AAV (AAV2/9-TRPV1-DTA), creating a model for tissue-specific ablation of sensory neurons (Fig. 1l). First, we performed intratracheal instillation of AAV2/9-TRPV1-DTA to target the local sensory fibers and specifically deplete neuron populations that innervate the lungs. In line with previous studies^42,50^, this approach resulted in selective depletion of sensory neurons from the vagal ganglia (Extended Data Fig. 1h), while somatosensory populations from DRG remained unaffected (Extended Data Fig. 1i). This selective depletion of lung-innervating vagal sensory neurons significantly reduced metastatic outgrowth in the mouse model of lung colonization (Fig. 1m-n). This effect was also observed when vagal sensory neurons were depleted through AAV2/9-TRPV1-DTA injected directly into the vagal ganglion (bilateral intra-ganglial injection) containing the cell bodies of lung-innervating neurons (Fig. 1o, Extended Data Fig. 1j-k), further confirming the involvement of vagal afferents during lung colonization by metastatic cancer cells. Immunostaining in the vagal ganglia revealed a progressive induction of Activating Transcription Factor 3 (ATF3) protein during lung colonization (Extended Data Fig. 1l), consistent with early engagement of lung-innervating sensory neurons by disseminated tumor cells and activation of a canonical neuronal injury-response pathway. Given the presence of cancer cell clusters adjacent to lung sensory fibers (Fig. 1c), we next investigated whether these metastatic cells possess the capacity to form direct interactions with vagal sensory neurons that may facilitate their survival and proliferation following extravasation into the lung.

### Nerve Injury–Induced Protein 1 (NINJ1) Is a Cancer–Neuron Crosstalk Mediator

**Extended Data Figure 2.**
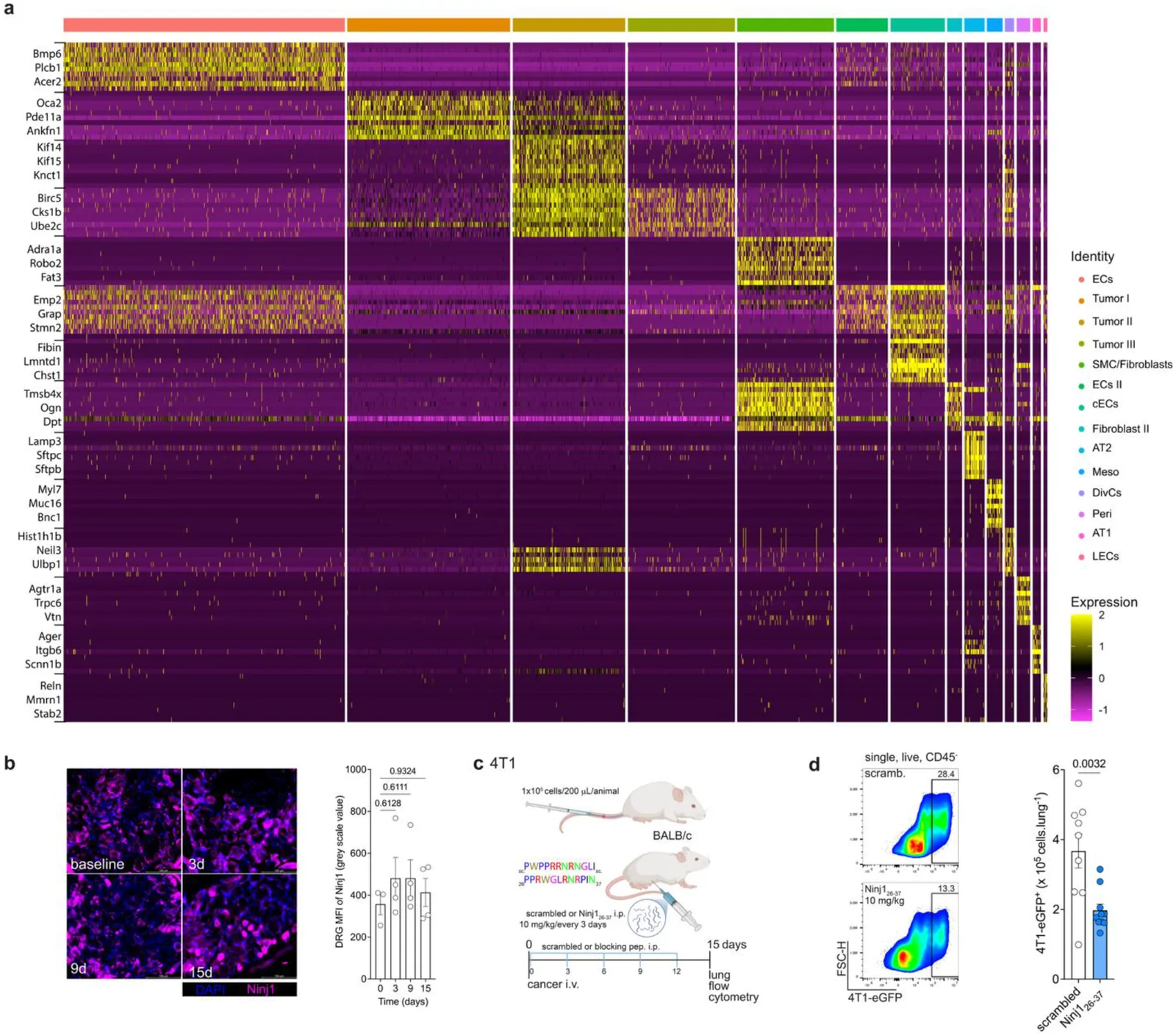
Neuronal and tumoral NINJ1 in tumor metastasis. **a.** Heatmap of top-expressed genes per non-immune cell cluster. **b.** Representative time-course immunohistochemistry (IHC) images and mean fluorescence intensity (MFI) quantification of NINJ1 in thoracic dorsal root ganglia. n = 3–4 mice per group. **c.** Experimental design for Ninj1-blocking peptide treatment, showing the model timeline and treatment schedule for NINJ1_26-37_ and scrambled peptide control in the 4T1 breast cancer model in Balb/c mice. **d.** Representative flow cytometry panels and quantification of tumor cells in lung tissue. n = 9 mice per group. Data are shown as mean ± SEM. Statistical significance was determined by one-way ANOVA with Tukey’s post hoc test (b), and unpaired two-tailed Student’s t-test in (d). i.v., intravenous injection; i.p., intraperitoneal injection; i.t., intratracheal instillation. LECs, lymphatic endothelial cells; ECs, endothelial cells; ECs II, endothelial cells II; cECs, circulating endothelial cells; AT1, alveolar type I cells; AT2, alveolar type II cells; Meso, mesothelial cells; Peri, pericytes;

**Figure 2.**
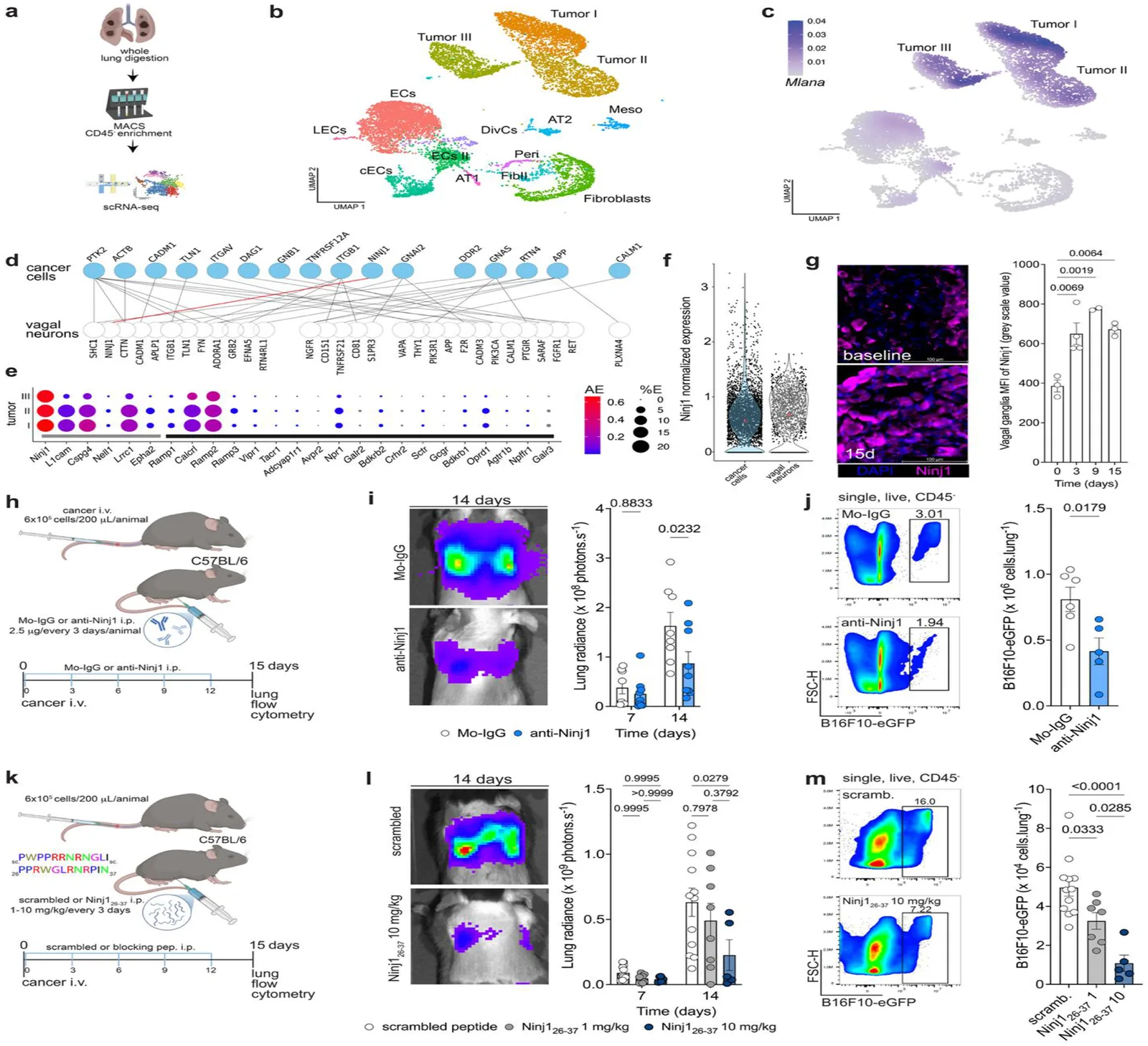
NINJ1 mediates tumor cell and neuron crosstalk. **a.** Schematic representation of tissue processing and cell enrichment for non-immune cells (CD45^−^), followed by single-cell RNA sequencing. **b.** Uniform manifold approximation and projection (UMAP) of sequenced CD45- cells. **c.** UMAP showing Mlana expression across the three melanoma clusters, designated Tumor I, Tumor II, and Tumor III. **d.** Predicted protein–protein interaction network between vagal sensory neurons (bottom, white) and tumor cells (top, blue), inferred from gene expression data using the OmniPath interaction database. The homophilic NINJ1–NINJ1 interaction is highlighted in red. **e.** Dot plot depicting the expression of genes related to potential neuronal adhesion mechanisms, including *Ninj1* (gray bar), together with neuropeptide receptor expression (black bar) in tumor cell clusters I, II, and III. **f.** Violin plot depicting normalized *Ninj1* expression in metastatic tumor cells and vagal sensory neurons from the merged single-cell RNA-seq analysis. Red diamonds indicate the mean expression for each cell type. **g.** Representative images of NINJ1 immunohistochemistry (IHC) at baseline and 15 days post-injection, with time-course quantification of mean fluorescence intensity in vagal sensory ganglia. n = 2–4 mice per group. **h.** Experimental design for NINJ1 antibody neutralization, showing the metastatic model timeline and treatment schedule. **i.** Representative BLI images and radiance quantification. n = 8–9 mice per group. **j.** Representative flow cytometry panels and quantification of tumor cells in lung tissue from mice treated with isotype control (Mo-IgG) or anti-NINJ1 neutralizing antibody. n = 5–6 mice per group. **k.** Experimental design for NINJ1-blocking peptide treatment, showing the metastatic model timeline and treatment schedule for Ninj1_26-37_ and scrambled peptide controls. **l.** Representative BLI images and radiance quantification. n = 5–12 mice per group. **m.** Representative flow cytometry panels and quantification of tumor cells in the lung tissue. n = 5-12 mice per group. Data are shown as mean ± SEM. Statistical significance was determined by Wilcoxon test (f), one-way ANOVA with Tukey’s post hoc test (g), two-way ANOVA (i and l), and unpaired two-tailed Student’s t-test (j and m). LECs, lymphatic endothelial cells; ECs, endothelial cells; ECs II, endothelial cells II; cECs, circulating endothelial cells; AT1, alveolar type I cells; AT2, alveolar type II cells; Meso, mesothelial cells; Peri, pericytes; FibII, fibroblasts II; i.v., intravenous injection; i.p., intraperitoneal injection; i.t., intratracheal instillation.

We proceeded to systematically map the potential mechanisms underlying cancer-neuron interactions by employing transcriptomics and protein-protein interaction analyses. For that, lung samples from mice bearing melanoma metastases (day 15) were initially processed using Magnetic Cell Separation Technology (MACS CD45 enrichment/depletion kit) to isolate the non-immune (CD45^-^) fraction containing tumor cells, followed by droplet-based (10x Genomics) library preparation and single-cell RNA-sequencing (Fig. 2a) as previously described ^29^. A total of 11670 cells were sequenced, and 14 clusters were annotated after uniform manifold approximation and projection (UMAP) (Fig. 2b, Extended Data Fig. 2a). Among those, three cancer cell clusters were identified based on *Mlana* (Melan-A) expression (Fig. 2c, Extended Data Fig. 2a), a canonical melanocytic differentiation antigen broadly expressed in primary and metastatic melanoma, ^51^ and annotated as Tumor I (enriched for melanocytic/MITF-high program genes e.g., *Sox10*, *Pmel*, *Dct*), Tumor II (enriched for cell-cycle genes e.g., *Mki67*, *Top2a*, *Ccnb1*, *Cenpf*), and Tumor III (enriched for cell-cycle genes, metabolic and biosynthetic program genes e.g., *Rrm2*, *Cks1b*, *Npm1*, *Gapdh*).

To infer candidate tumor-neuron interactions, we integrated our metastatic tumor cell scRNA-seq dataset with a single-cell transcriptomic atlas of vagal sensory neurons (GSE124312). We performed computational protein-protein interaction analysis using the OmniPath database ^52^. This approach allowed us to predict potential protein-protein interactions between the two cell types based on the co-expression of known interacting molecule pairs from the OmniPath database. The analysis revealed a network of multiple potential interactions between the two cells (Fig. 2d). Among these, nerve injury-induced protein 1 (*Ninj1*) NINJ1↔NINJ1 emerged as one of the top-scoring interactions (OmniPath interaction identifier 117825) and the only homophilic adhesion pair (cohesion) in the top decile of the ranked list (Fig. 2d). Our analyses showed that *Ninj1* expression was high in all cancer cell clusters and surpassed that of neuropeptide receptors, including *Ramp1* and *Calcrl* recently identified in melanoma (Fig. 2e). In accordance with the prediction algorithm, *Ninj1* transcripts were observed in 77.9% of cancer cells at levels comparable to those found in vagal sensory neurons (mean normalized expression; Wilcoxon test, P = 0.38; detected in 80.6% of neurons) (Fig. 2f**, Supplementary Table 1**).

NINJ1 is a 16-kDa cell adhesion protein containing two hydrophobic transmembrane domains, which anchor it to the plasma cell membrane, and two extracellular regions at the C- and N-termini, containing an N-terminal adhesion motif (N-NAM) responsible for mediating the homophilic binding (cohesion) to another NINJ1 molecule on an adjacent cell. ^53-55^ NINJ1 was initially identified in sensory neurons and Schwann cells as an injury-response molecule that promotes neurite outgrowth in cultured sensory neurons and axonal repair after nerve injury, playing critical roles in nerve regeneration. ^53-55^ We hypothesized that metastatic cells may exploit this repair-associated molecule to engage vagal sensory neurons upon arrival in the lung. Consistent with this idea, lung colonization also induced a marked increase in NINJ1 protein expression in vagal ganglion neurons (Fig. 2g), while thoracic dorsal root ganglia did not show a corresponding increase (Extended Data Fig. 2b). Given that this upregulation was accompanied by induction of ATF3 as noted before (Extended Data Fig. 1l), a canonical marker of neuronal stress and injury responses, these observations indicate an activation of a neural repair program specifically within vagal afferents during metastasis. Together, these findings suggest that the injury-mimicking characteristics of cancer cells trigger repair-associated pathways in vagal sensory neurons, positioning NINJ1 as a bidirectional molecule with the potential to facilitate direct neuron–cancer communication in the metastatic microenvironment.

**Extended Data Figure 3.**
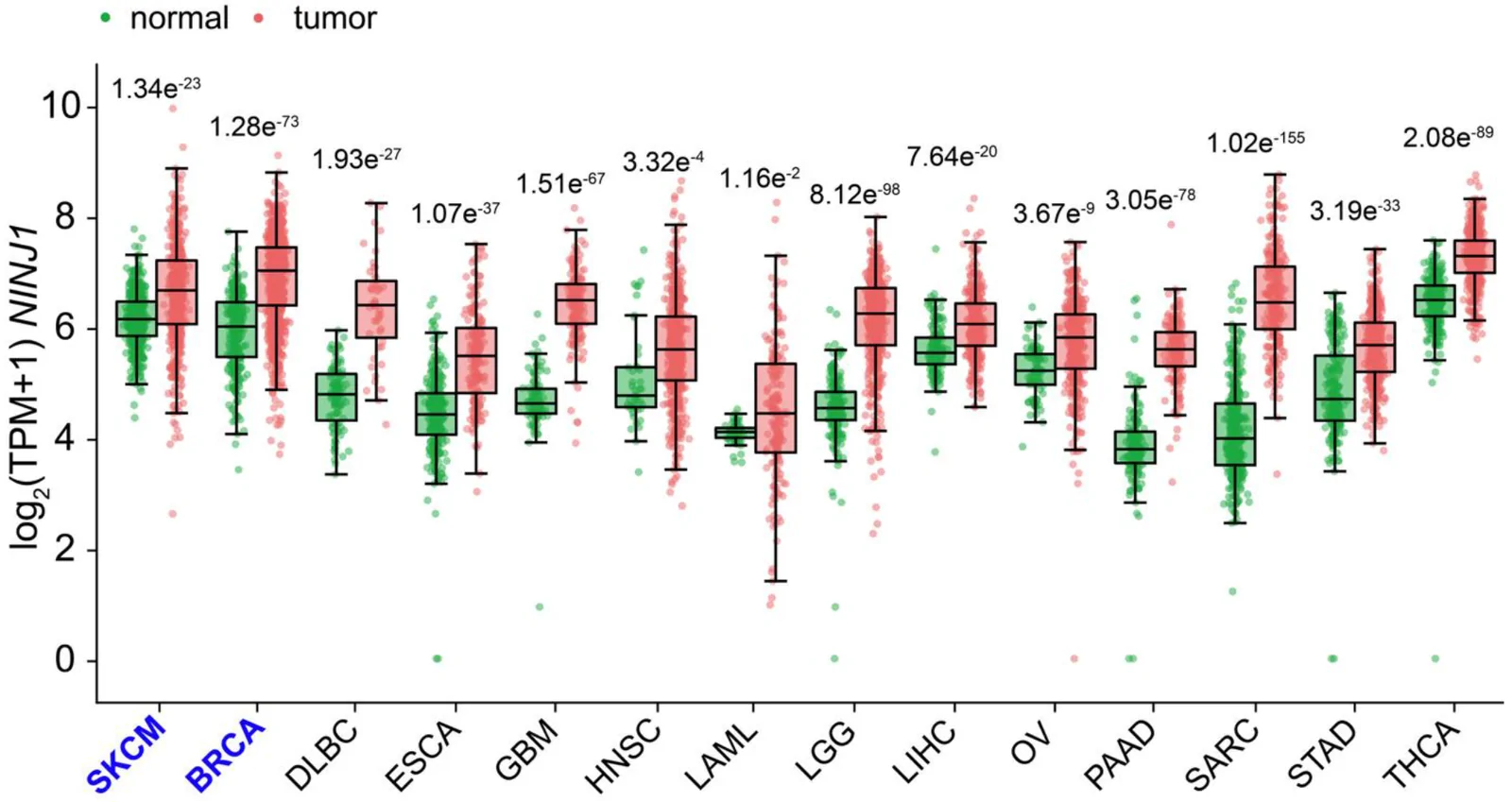
*NINJ1* expression is increased in tumors compared to normal tissues across multiple human cancer types. Analysis of NINJ1 mRNA expression in human tumors versus corresponding normal tissues using data from TCGA, as accessed through GEPIA3^82^. Cancer types included skin cutaneous melanoma (SKCM), breast invasive carcinoma (BRCA), diffuse large B-cell lymphoma (DLBC), esophageal carcinoma (ESCA), glioblastoma multiforme (GBM), head and neck squamous cell carcinoma (HNSC), acute myeloid leukemia (LAML), brain lower-grade glioma (LGG), liver hepatocellular carcinoma (LIHC), ovarian serous cystadenocarcinoma (OV), pancreatic adenocarcinoma (PAAD), sarcoma (SARC), stomach adenocarcinoma (STAD), and thyroid carcinoma (THCA). Box plots show tumor and normal tissue expression levels, with statistical significance calculated in GEPIA3.

**Extended Data Figure 4.**
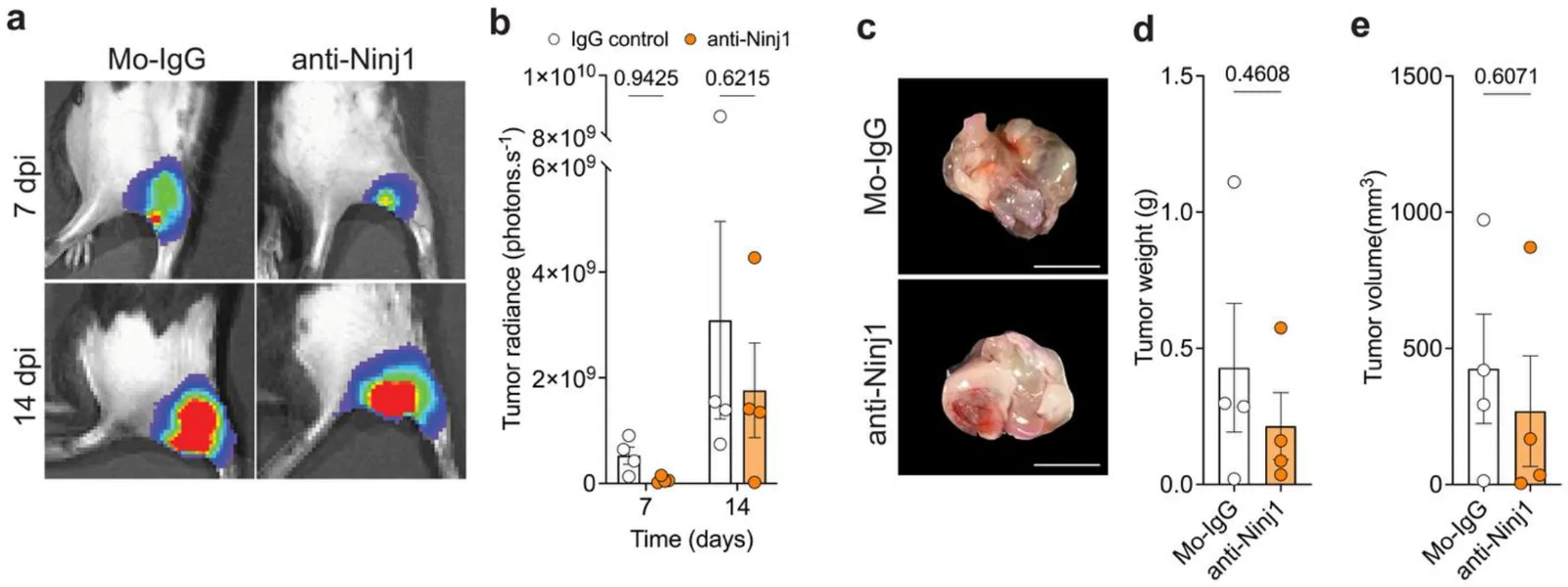
NINJ1 antibody neutralization does not reduce orthotopic B16F10 melanoma outgrowth. **a.** Representative bioluminescence imaging (BLI) images of Mo-IgG- and anti-NINJ1-treated mice over time. **b.** Quantification of BLI radiance in orthotopic tumors over time. n = 4 mice per group. **c.** Representative images of dissected orthotopic tumors. **d.** Orthotopic melanoma tumor weight. n = 4 mice per group. **e.** Orthotopic melanoma tumor volume. n = 4 mice per group. Data are shown as mean ± SEM. Statistical significance was determined by two-way ANOVA (b) and unpaired two-tailed Student’s t-test (d and e). Mo-IgG, mouse IgG control; dpi, days post-injection.

To test the functional role of NINJ1 in metastasis, we first inhibited NINJ1 homophilic interactions pharmacologically. Mice bearing B16F10 metastases were treated every three days with either a neutralizing anti-NINJ1 antibody or an isotype control (Fig. 2h-j). In a parallel approach, mice received a custom-designed NINJ1 blocking peptide (NINJ1_26-37_), a synthetic 12-amino acid sequence complementary to residues 26-37 from NINJ1’s cohesion epitope (N-NAM), while the control group was treated with a scrambled peptide (Fig. 2k-m). Both pharmacological strategies aimed at inhibiting NINJ1 homophilic interactions significantly reduced melanoma metastatic burden in the lungs compared with their respective controls (Fig. 2i-j, l-m). Blocking NINJ1 also reduced lung colonization by 4T1 breast cancer cells in BALB/c mice (Extended Data Fig. 2c-d), indicating that the pro-metastatic role of NINJ1 extends beyond a single cancer type. Analysis of public human transcriptomic datasets confirmed that *NINJ1* mRNA is elevated in several cancers relative to normal tissues (Extended Data Fig. 3), including skin cutaneous melanoma (SKCM) and breast cancer (BRCA), indicating the potential clinical relevance of this mechanism. This is in line with recent reports linking increased NINJ1 expression to tumor progression and metastasis in non-small cell lung cancer and prostate cancer. ^56,57^ By contrast, NINJ1 inhibition did not substantially protect mice in an orthotopic melanoma model in which B16F10 cells were injected into the skin, a tissue predominantly innervated by DRG somatosensory neurons rather than vagal afferents (Extended Data Fig. 4a-e). These data suggest that NINJ1 may be particularly important in metastatic colonization of internal organs innervated by the vagus nerve.

**Extended Data Figure 5.**
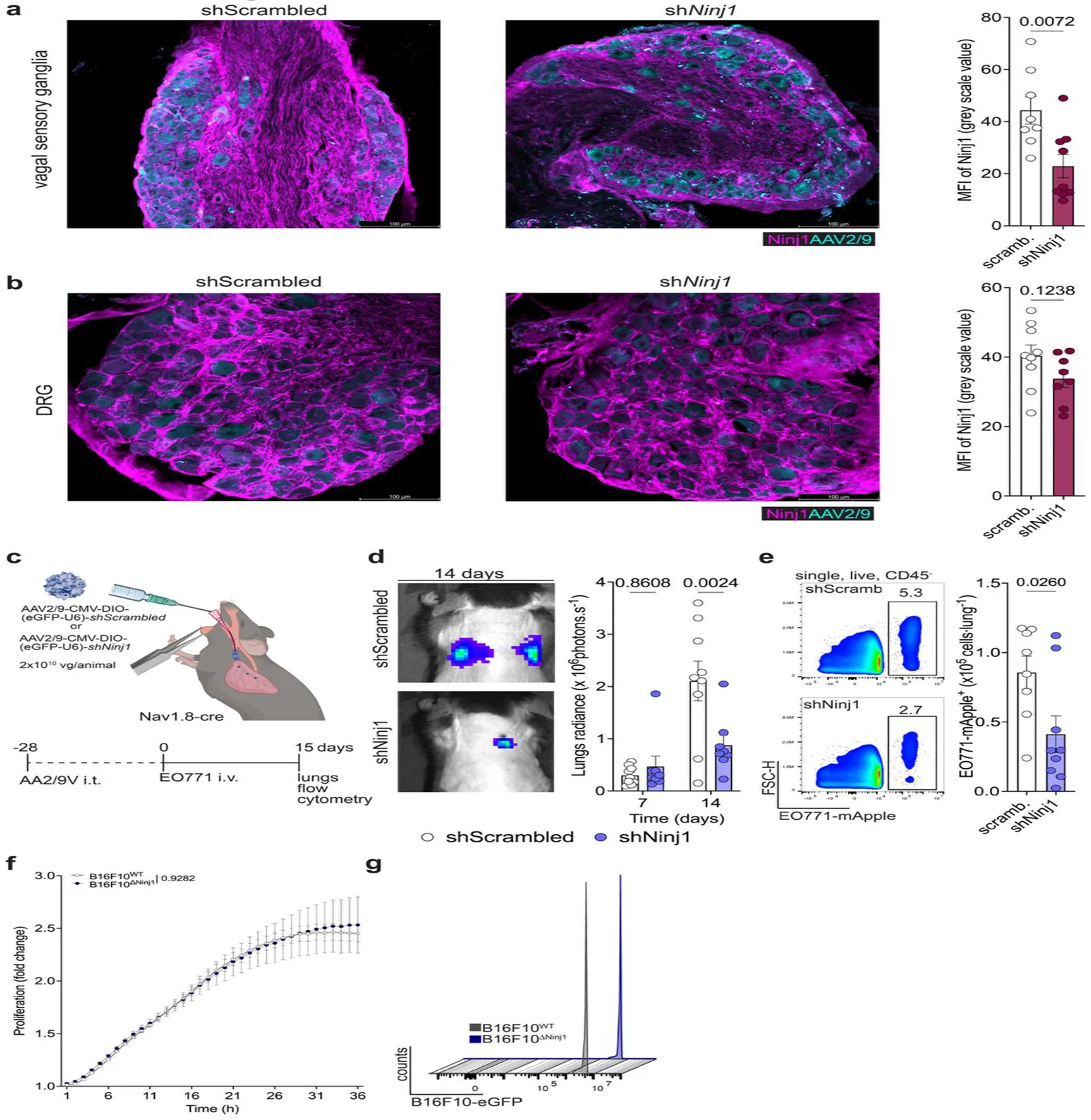
Additional validation of NINJ1 knockdown and knockout. **a.** Representative images and quantification of NINJ1 mean fluorescence intensity (MFI) in vagal sensory ganglia, validating the efficiency and specificity of NINJ1 knockdown following intratracheal instillation of AAV2/9-CMV-DIO-(eGFP-U6)-short hairpin constructs. n = 8-9 mice per group. **b.** Representative images and quantification of NINJ1 MFI in dorsal root ganglia (DRG), validating the efficiency and specificity of NINJ1 knockdown following intratracheal instillation of AAV2/9-CMV-DIO-(eGFP-U6)-short hairpin constructs. n = 8-9 mice per group. **c.** Experimental design for neuronal *Ninj1* knockdown using Cre-dependent AAV, followed by EO771-mApple tumor cell injection, including model timeline and vector information **d.** Representative bioluminescence imaging (BLI) images and radiance quantification. n = 8-9 mice per group **e.** Representative flow cytometry panels and quantification of tumor cells in lung tissue. n = 8-9 mice per group. **f-g**. Proliferation curve (f) and eGFP intensity (g) for B16F10^WT^ and B16F10^ΔNinj1^. Data are shown as mean ± SEM. Statistical significance was determined by unpaired two-tailed Student’s t-test (a, b, e, and g), and two-way ANOVA (d and f). i.t., intratracheal instillation; i.v., intravenous injection; sh, short hairpin.

**Figure 3.**
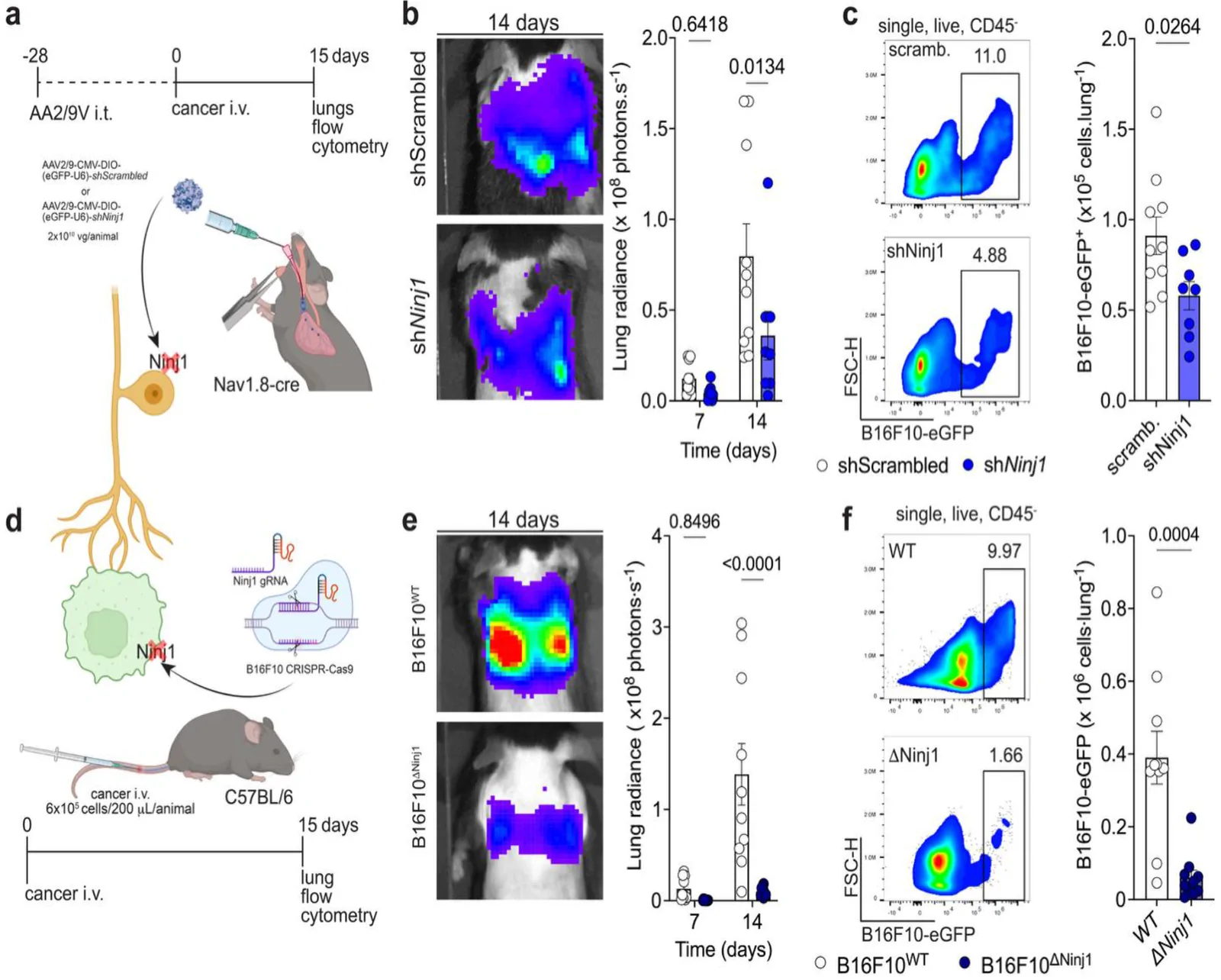
Disruption of NINJ1 expression in neurons or tumor cells decreases cancer metastasis. **a.** Experimental design for neuronal knockdown of *Ninj1* using Cre-dependent AAV, including the metastatic model timeline and vector information. **b.** Representative BLI images and radiance quantification. n = 8–10 mice per group. **c.** Representative flow cytometry panels and quantification of tumor cells in lung tissue. n = 8–10 mice per group. **d.** Experimental design for CRISPR–Cas9-mediated *Ninj1* knockout in tumor cells and metastatic model timeline. **e.** Representative BLI images and radiance quantification. n = 10 mice per group. **f.** Representative flow cytometry panels and quantification of tumor cells in lung tissue. n = 10 mice per group. Data are shown as mean ± SEM. Statistical significance was determined by two-way ANOVA (b and e), and unpaired two-tailed Student’s t-test (c and f). i.v., intravenous injection; i.t., intratracheal instillation; sh, short hairpin.

Next, we addressed the specific role of NINJ1 expressed by vagal sensory neurons in metastatic outgrowth. For that, we targeted neuronal *Ninj1* using an Adeno-associated virus (AAV) carrying the Cre-dependent short hairpin RNA targeting *Ninj1* (AAV2/9-DIO-eGFP-sh*Ninj1*) or the scrambled-shRNA as a control. The AAVs were delivered intratracheally in Nav1.8-Cre^+/−^ mice to knockdown *Ninj1* expression specifically in lung-innervating vagal sensory neurons (Fig. 3a), which was confirmed by a significant reduction in the expression of NINJ1 protein observed in the vagal ganglion (Extended Data Fig. 5a) but not in thoracic DRG neurons (Extended Data Fig. 5b). Neuronal *Ninj1* knockdown led to a marked reduction in lung colonization by both metastatic melanoma (Fig. 3b-c) and breast cancer cells (Extended Data Fig. 5c-e), confirming the involvement of neuronal NINJ1 during lung colonization by metastatic cells. Lastly, we assessed the role of NINJ1 expressed by cancer cells. To accomplish this, CRISPR-Cas9 technology was utilized in conjunction with a synthetic guide RNA (gRNA) specifically designed to target the N-NAM sequence within the extracellular adhesion motif of *Ninj1*, resulting in a loss-of-function disruption of the NINJ1 gene as verified by genomic sequencing. This allowed us to generate NINJ1-deficient clones (ΔNinj1) from the cancer cell lines used in our study (Fig. 3d**, Supplementary Table 2**). Clones selected for subsequent studies exhibited normal *in vitro* proliferation rates, eGFP fluorescence intensity, and STR profiles comparable to those of parental wild-type cells (Extended Data Fig. 5f-g, **Supplementary Table 3**). NINJ1-deficient cells displayed a markedly reduced capacity to colonize the lung relative to wild-type controls (Fig. 3e-f). These findings demonstrate that NINJ1 is not simply associated with disease progression but is functionally required on both sides of the neuron–tumor interface to enable the full metastatic potential of lung-colonizing cancer cells.

### NINJ1 Reprograms Metastatic Cancer Cells with Enhanced Proliferative Capacity.

**Figure 4.**
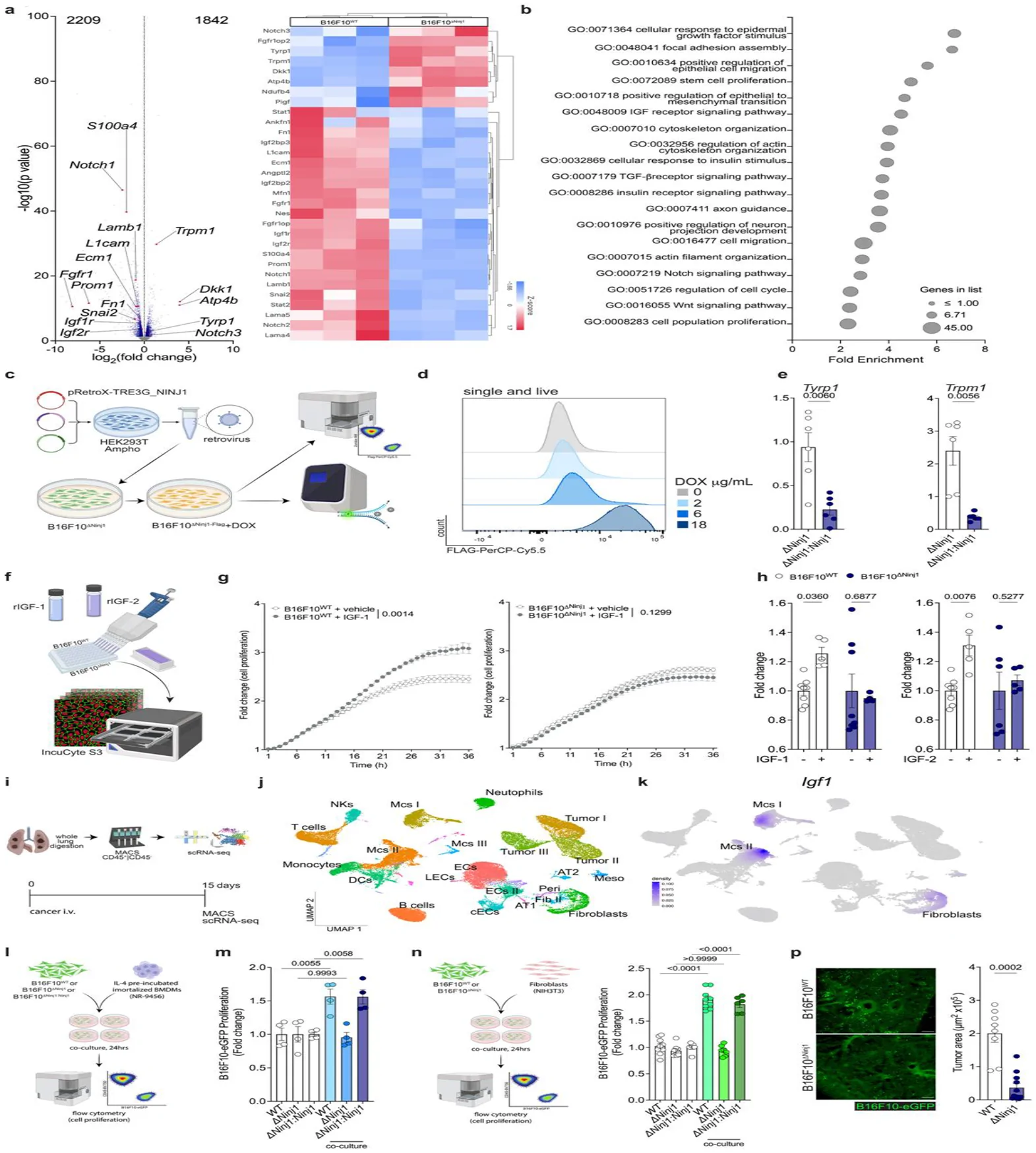
NINJ1 confers cancer stem-like signature and response to environmental proliferative cues. **a**. volcano plot and heatmap of differentially expressed genes (DEGs) in B16F10^Δ^Ninj1 versus B16F10^WT^ cells; The heatmap highlights upregulation of melanocyte differentiation markers and the downregulation of stemness markers and growth factor receptors. n = 3 per group. **b.** Gene Ontology (GO) analysis of genes downregulated in B16F10^Δ^Ninj1_._ **c.** Schematic representation of doxycycline (DOX)-inducible FLAG^®^-NINJ1 expression in B16F10^ΔNinj1^, hereafter referred to as B16F10^Δ^Ninj1:Ninj1_._ **d.** Flow cytometry histogram validating B16F10^ΔNinj1:Ninj1^ cells using an anti-DYKDDDDK (FLAG^®^) antibody. **e.** mRNA expression profiles of *Tyrp1* and *Trpm1* in B16F10^ΔNinj1^ and B16F10^Δ^Ninj1:Ninj1 cells. **f.** Schematic representation of the proliferation assay pipeline testing recombinant growth factors in WT and KO cells using the IncuCyte S3 platform. **g**. Proliferation curves for B16F10^WT^ and B16F10^ΔNinj1^ treated or not with recombinant insulin-like growth factor 1 (rIGF-1). n = 4 per group. **h**. Fold change in proliferation of B16F10^WT^ and B16F10^ΔNinj1^ treated or not with recombinant insulin-like growth factor 1 or 2 (rIGF-1 or rIGF-2). n = 5-8 per group. **i**. Schematics representation of tissue processing, cell enrichment, and single-cell RNA sequencing (scRNA-seq). **j**. UMAP of immune (CD45+) and non-immune (CD45−) cells from lungs collected 15 days after tumor injection in C57Bl/6 mice. **k**. UMAP showing *Igf1* expression density, with higher expression in macrophages and fibroblasts. **l.** schematic representation of B16F10^WT^, B16F10^ΔNinj1^, or B16F10^ΔNinj1:Ninj1,^ cell co-cultured with pro-regenerative macrophages (M2-like). B16F10^ΔNinj1:Ninj1^ cells were also co-incubated with doxycycline 18 μg/mL. **m.** Flow cytometry quantification of tumor cell proliferation in monoculture, or co-cultured with pro-regenerative macrophages. n = 4 per group. **n.** Schematic representation of B16F10^WT^, B16F10^ΔNinj1^, or B16F10^Δ^Ninj1:Ninj1 ce_ll_s co-cultures with NIH3T3 fibroblasts. B16F10^ΔNinj1:Ninj1^ cells were also co-incubated with doxycycline 18 μg/mL. **o**. Flow cytometry quantification of tumor cell proliferation in monoculture, or co-cultured with fibroblasts. n = 6-9 per group. **p.** Representative images and quantification of B16F10-eGFP cells, measured as eGFP^+^ area, in precision-cut lung slices (PCLS) seeded with B16F10^WT^ or B16F10^ΔNinj1^ cells. n = 4 replicates; n = 3 mice per group and 8–11 random fields for N. Data are shown as mean ± SEM. Statistical significance was determined by unpaired two-tailed Student’s t-test (e and p), two-way ANOVA (g and h), and one-way ANOVA with Tukey’s post hoc test (m and o). NKs, natural killer cells; Mcs I, alveolar macrophages; Mcs II, interstitial macrophages; DCs, dendritic cells; Mcs III, macrophages III; LECs, lymphatic endothelial cells; ECs, endothelial cells; ECs II, endothelial cells II; cECs, circulating endothelial cells; AT1, alveolar type I cells; AT2, alveolar type II cells; Meso, mesothelial cells; Peri, pericytes; FibII, fibroblasts II; Igf1, insulin-like growth factor 1; IL-4, interleukin-4; BMDMs, bone marrow-derived macrophages; RTX, resiniferatoxin.

We next sought to define the molecular mechanisms by which NINJ1 confers such a profound metastatic advantage. To determine whether NINJ1 exerts any influence on the expression of specific genes and transcriptional programs of metastatic cells, we performed RNA sequencing to compare the transcriptomes of wild-type B16F10 melanoma cells with their isogenic NINJ1-deficient counterparts (ΔNinj1). This unbiased analysis revealed a sweeping transcriptional reprogramming, with thousands of genes differentially expressed as a function of NINJ1 status (Fig. 4a). Loss of NINJ1 shifted metastatic cells away from a stem-like, undifferentiated state: genes associated with stemness and neural repair programs, including *Prom1* and *L1cam*, were reduced in ΔNinj1 cells, whereas differentiation markers such as *Trpm1* and *Tyrp1* were increased (Fig. 4a). Gene Ontology analysis of the NINJ1-dependent gene set further supported this phenotypic shift, revealing enrichment for biological processes central to tumor progression, including positive regulation of epithelial to mesenchymal transition, Wnt signaling pathway, stem cell proliferation, regulation of cell cycle, and response to growth factors (Fig. 4b), along with pathways linked to well-established functions of NINJ1 in nerve repair, such as promoting neuron projection development and guiding axons (Fig. 4b). Importantly, rescuing the ability of ΔNinj1 cells to express NINJ1 using a doxycycline-inducible NINJ1 system was sufficient to suppress the expression of differentiation markers once again (Fig. 4c-e), confirming the specificity of the ΔNinj1 clones generated in our study. These results indicate that NINJ1 acts as a transcriptional regulator of pro-regenerative pathways required for cancer cells to maintain a dedifferentiated, plastic transcriptional state known to boost metastatic potential, therapy resistance, and adaptation to new tissues.

**Extended Data Figure 6.**
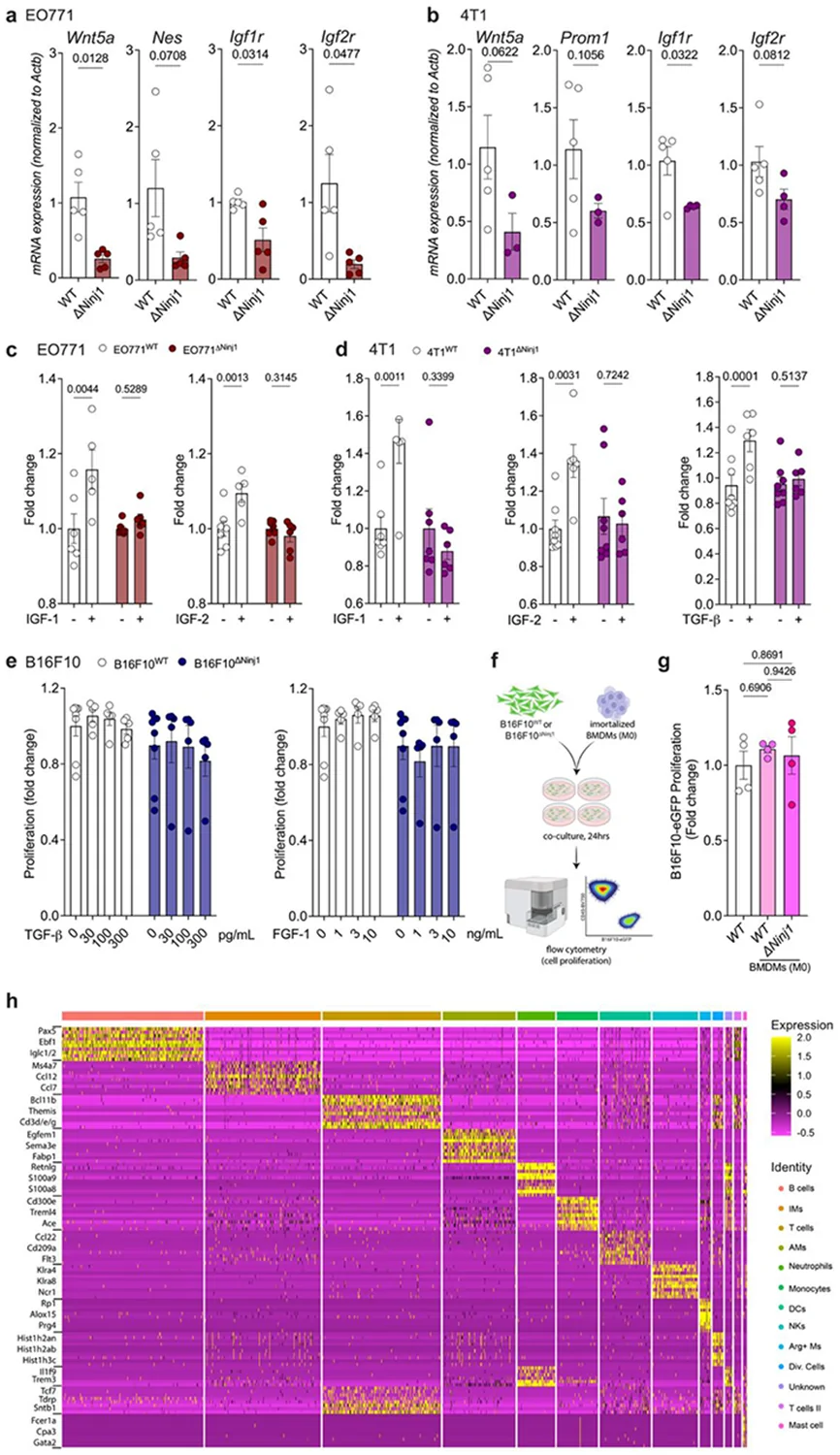
Additional characterization of NINJ1-mediated reprogramming. **a.** Expression profile of stemness markers (*Wnt5a* and *Nes*) and growth factor receptors (*Igf1r* and *Igf2r*) in EO771^WT^ and EO771^ΔNinj1^. **b.** Expression profile of stemness markers (*Wnt5a* and *Prom1*) and growth factor receptors (Igf1r and Igf2r) in 4T1^WT^ and 4T1^ΔNinj1^. **c.** Fold change in proliferation of EO771^WT^ and EO771^ΔNinj1^ cells treated or not with recombinant insulin-like growth factor 1 (rIGF-1) or rIGF-2. n = 5-8 per group. **d.** Fold change in proliferation of 4T1^WT^ and 4T1^ΔNinj1^ cells treated or not with recombinant insulin-like growth factor 1 (rIGF-1), rIGF-2, or recombinant TGF-β (rTGF-β). n = 5-8 per group. **e.** Fold change in proliferation of B16F10^WT^ and B16F10^ΔNinj1^ cells in response to concentration gradients of rTGF-β and recombinant fibroblast growth factor-1 (rFGF-1). n = 5 per group. **f.** Schematic representation of B16F10^WT^ and B16F10^ΔNinj1^ cells co-cultured with non-polarized BMDMs (M0-like). **g.** Fold change in proliferation of B16F10^WT^ and B16F10^ΔNinj1^ co-cultured with M0-like BMDMs. **h.** Heatmap of top-expressed genes per cluster of immune cells. Data are shown as mean ± SEM. Statistical significance was determined by unpaired two-tailed Student’s t-test (a and b), two-way ANOVA (c, d, and e), and one-way ANOVA with Tukey’s post hoc test (g).

Of particular relevance to post-extravasation growth during lung colonization, our analysis revealed that NINJ1 expression transcriptionally primes metastatic cancer cells to better sense and respond to mitogenic signals in the new tissue environment. NINJ1 was required to maintain high expression levels of key growth factor receptors, including the insulin-like growth factor receptors *Igf1r* and *Igf2r*, fibroblast growth factor receptor *Fgfr1*, and transforming growth factor receptor *Tgfbr2* (Fig. 4a-b). This NINJ1-dependent regulation of growth factor receptors was conserved across both melanoma and breast cancer cell lines (Extended Data Fig. 6a-b), suggesting a generalizable mechanism that may sensitize cells to growth-inducing factors. To test this hypothesis functionally, we assessed the proliferative response of WT and ΔNinj1 cells to recombinant insulin-like growth factors (IGF-1 and IGF-2), potent mitogens known to be abundant in the tumor microenvironment and critical for cancer progression. Live-cell imaging confirmed that all examined cancer cell types, including melanoma and breast cancer lines, demonstrated a significant increase in proliferation following stimulation with IGF-1 or IGF-2. This proliferative response was mediated by NINJ1, as ΔNinj1 cells did not respond to these factors (Fig. 4f-h, Extended Data Fig. 6c-d). Importantly, other growth factors tested, such as FGF-1 and TGF-β, failed to produce similar generalizable effects across the different cancer cell types despite expressing receptors for them: FGF-1 and TGF-β did not stimulate proliferation in B16F10 melanoma cells, whereas 4T1 breast cancer cells responded to TGF-β through an NINJ1-dependent mechanism (Extended Data Fig. 6d-e). These differences, together with the observation that WT and ΔNinj1 cells exhibit similar basal proliferation in nutrient-rich conditions, indicate that the reduced proliferative capacity of NINJ1-deficient cancer cells is not due to general defects in metabolism or cell division. Rather, these data provide functional evidence that NINJ1 regulates cancer cell responsiveness to specific growth factors.

**Extended Data Figure 7.**
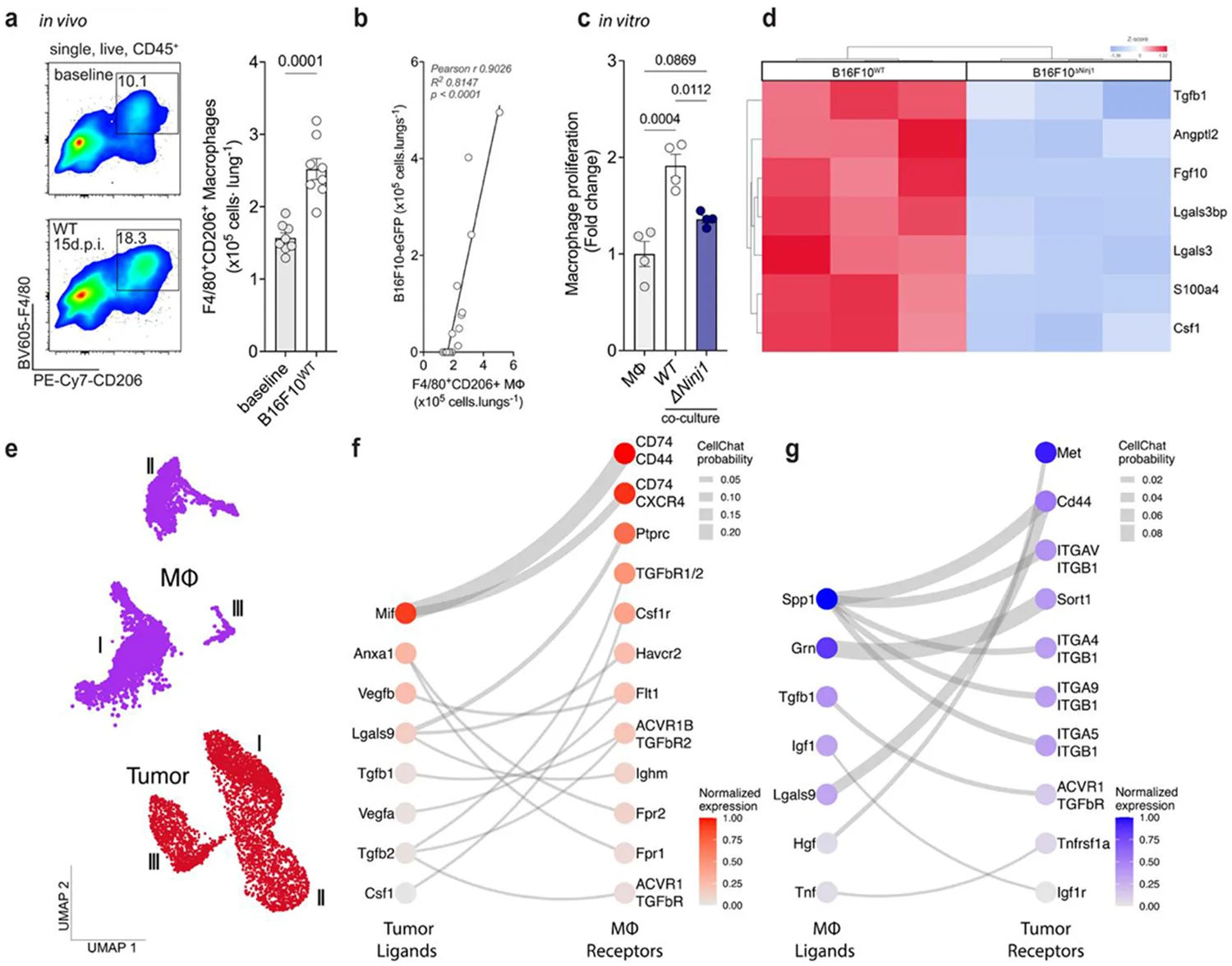
NINJ1-dependent tumor–macrophage crosstalk supports macrophage proliferation/expansion. **a.** Quantification of macrophage proliferation *in vitro* in monoculture or following co-culture with B16F10^WT^ or B16F10^ΔNinj1^ cells. **b.** Heatmap showing the expression of macrophage-regulatory and growth factor-related genes in B16F10^WT^ or B16F10^ΔNinj1^ cells. **c.** Representative flow cytometry plots and quantification of F4/80^+^CD206^+^ macrophages in lung tissue at baseline and 15 days after B16F10^WT^ tumor cell injection. **d.** Pearson correlation between the number of B16F10-eGFP^+^ tumor cells and F4/80^+^CD206^+^ macrophages in lung tissue. **e.** UMAP visualization of macrophage (MΦ), and tumor cell clusters identified by single-cell RNA sequencing of lung metastases. Macrophage clusters are shown in purple and tumor clusters in red. **f.** Predicted ligand–receptor interaction network from tumor cells to macrophages, based on CellChat analysis. Node color indicates normalized gene expression, and edge thickness represents interaction probability. **g.** Predicted ligand–receptor interaction network from macrophages to tumor cells, based on CellChat analysis. Node color indicates normalized gene expression, and edge thickness represents interaction probability. Data are shown as mean ± SEM. Statistical significance was determined by one-way ANOVA (a), Wald test with Benjamini–Hochberg correction for multiple comparisons (b), Unpaired two-tailed Student’s t-test (c), Pearson correlation coefficient (d), and CellChat permuting testing (f and g). MΦ, macrophages; dpi, days post-injection.

After establishing that NINJ1 enables cancer cells to respond to IGFs, we proceeded to verify the production of these factors within the metastatic niche through scRNA-seq analysis of immune and non-immune cell populations in the lungs of mice with melanoma metastases (Fig. 4i). Although *Igf2* transcripts were undetectable, the analysis confirmed the presence of *Igf1* in the lung tissue, with macrophages and fibroblasts identified as the primary sources (Fig. 4i-k). We then proceeded to test whether NINJ1-dependent program was also essential for metastatic cells to exploit the growth support offered by these lung resident cell populations. We first co-cultured WT and ΔNinj1 cancer cells with bone marrow-derived macrophages (BMDMs) previously polarized into a pro-regenerative phenotype (M2-like), a subtype known to promote tissue repair and express high levels of IGF-1^58,59^. Consistent with our findings from IGF treatments, the presence of macrophages markedly enhanced the proliferation of cancer cells expressing NINJ1 (WT), and this effect was lost in NINJ1-deficient clones (ΔNinj1) (Fig. 4l-m). Similarly, the presence of fibroblasts also increased the proliferation of cancer cells in a NINJ1-dependent manner (Fig. 4n-o). Reintroduction of NINJ1 expression in ΔNinj1 cells completely restored their capacity to respond to growth-promoting signals from co-cultured macrophages (Fig. 4m) and fibroblasts (Fig. 4o). The specificity of this effect was further emphasized by the observation that non-polarized (M0) macrophages, which exhibit lower IGF-1 expression^58,59^, did not confer a comparable proliferative benefit (Extended Data Fig. 6f-g). Loss of NINJ1 also reduced the proliferation of cancer cells in *ex vivo* lung slices (Fig. 4p), further corroborating the role of NINJ1 as an important factor aiding the ability of metastatic cancer cells to overcome the post-extravasation bottleneck and proliferate within novel tissue environments. Notably, NINJ1^+^ cancer cells reciprocally remodeled the macrophage compartment, promoting the expansion of lung-resident macrophage populations *in vivo* (F4/80^+^CD206^+^ and F4/80^+^CD11c^+^) (Extended Data Fig. 7a-b) and stimulating the proliferation of macrophages *in vitro* (Extended Data Fig. 7c-d). Multiparametric flow cytometric analysis of the lung immune landscape also highlighted the concordance between macrophage abundance and metastatic burden across all experimental strategies disrupting this neuro-metastatic axis: although depletion of sensory neurons did not alter the baseline immune composition of the lung (**Supplementary Data Fig. 2)**, macrophage populations were consistently reduced in parallel with cancer cells (Fig. 1-3) following disruption of sensory neurons (**Supplementary Data Fig. 3–8**) or of NINJ1 (**Supplementary Data Fig. 9–14**). These findings indicate that the observed changes in macrophage abundance might arise as a consequence of cancer cell–immune interactions downstream of neural engagement and NINJ1 signaling, rather than from direct neuro–immune regulation. Consistent with this interpretation, metastatic cancer cells expressed multiple macrophage-regulatory factors, some of which were driven by NINJ1, such as MCSF (*Csf1*) (Extended Data Fig. 7d-f), providing a plausible mechanism for direct control of macrophage expansion and reinforcing the existence of a cancer-initiated feedback loop that is distinct from recently described neuroimmune pathways involved in immune evasion. These findings indicate that NINJ1 not only enables cancer cells to benefit from macrophage-derived trophic signals but also supports a reciprocal circuit in which metastatic cells foster the accumulation of macrophage populations with pro-regenerative features.

### NINJ1 Couples Neuron–Cancer Interaction to β-Catenin–Dependent Metastatic Fitness

**Extended Data Figure 8.**
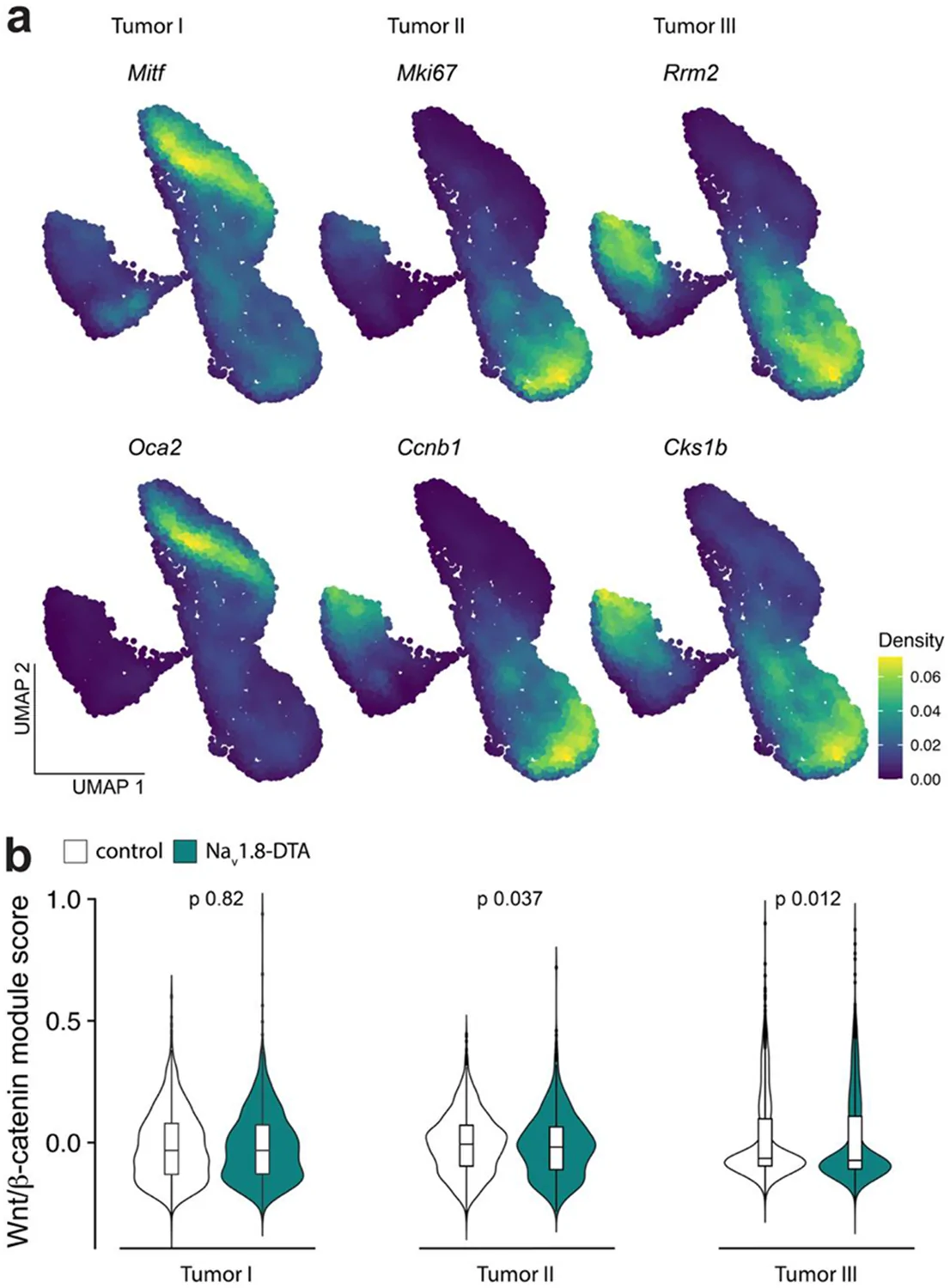
Nav1.8^+^ sensory neuron depletion reduces Wnt/β-catenin module score in metastatic tumor cells. **a.** Expression density of tumor cluster markers: Tumor I, *Mitf* and *Oca2*; Tumor II, *Mki67* and *Ccnb1*; and Tumor III, *Rrm2* and *Cks1b*. **b.** Wnt/β-catenin module score in tumor clusters from control and Na_v_1.8-DTA mice. Data are shown as violin plots of the Wnt/β-catenin module score per cell. Statistical significance was determined by a two-sided Wilcoxon rank-sum test with Benjamini–Hochberg correction. Tumor I n = 1,998 control and 1,737 Nav1.8-DTA cells; Tumor II, n = 1,382 control and 1,199 Nav1.8-DTA cells; Tumor III, n = 1,312 control and 1,111 Nav1.8-DTA cells.

**Extended Data Figure 9.**
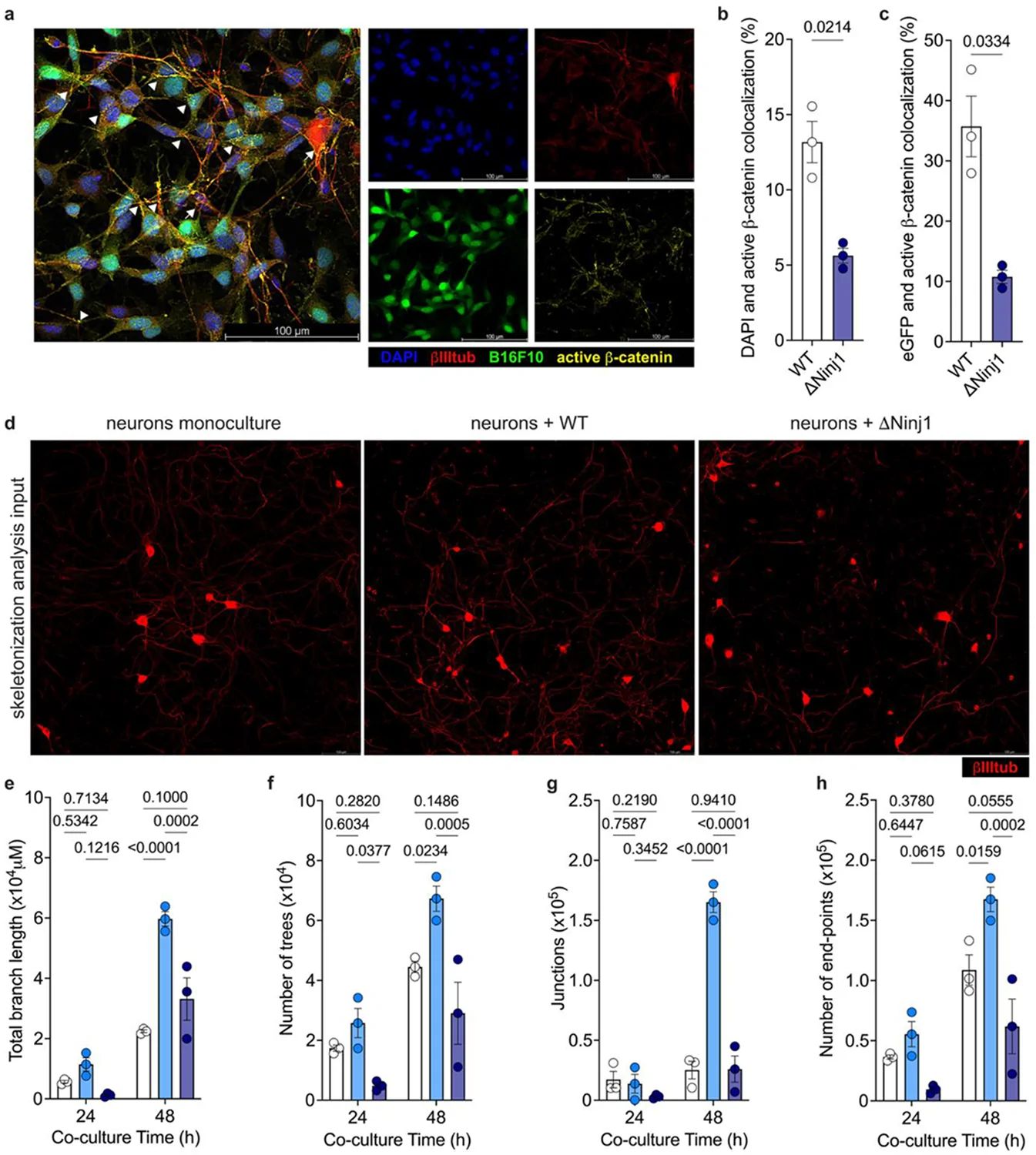
Tumor-derived NINJ1 promotes neurite outgrowth and increases network complexity. **a.** Representative image of active β-catenin cellular compartmentalization. Arrowheads indicate tumor cells, and arrows indicate sensory neurons with dense active β-catenin signal. **b-c.** Quantification of active β-catenin colocalization with DAPI-labeled nuclei (b) and eGFP^+^ tumor cells. n = 3 per group. **d**. Representative input images for skeletonization analysis of vagal sensory neurons sprouting, accessed by β-tubulin III staining, in monoculture or in co-culture with B16F10^WT^ or B16F10^Δ^Ninj1 ce_ll_s_._ **e**. Quantification of neuronal total branch length over time. n = 3 per group per time point. **f**. Quantification of the number of neuronal trees over time. n = 3 per group per time point. **g.** Quantification of the number of junctions over time. n = 3 per group per time point. **h.** Quantification of the number of neuronal endpoints over time. n = 3 per group per time point. Data are shown as mean ± SEM. Statistical significance was determined by unpaired two-tailed Student’s t-test (b and c), and two-way ANOVA with Tukey’s post hoc test (e – h).

**Figure 5.**
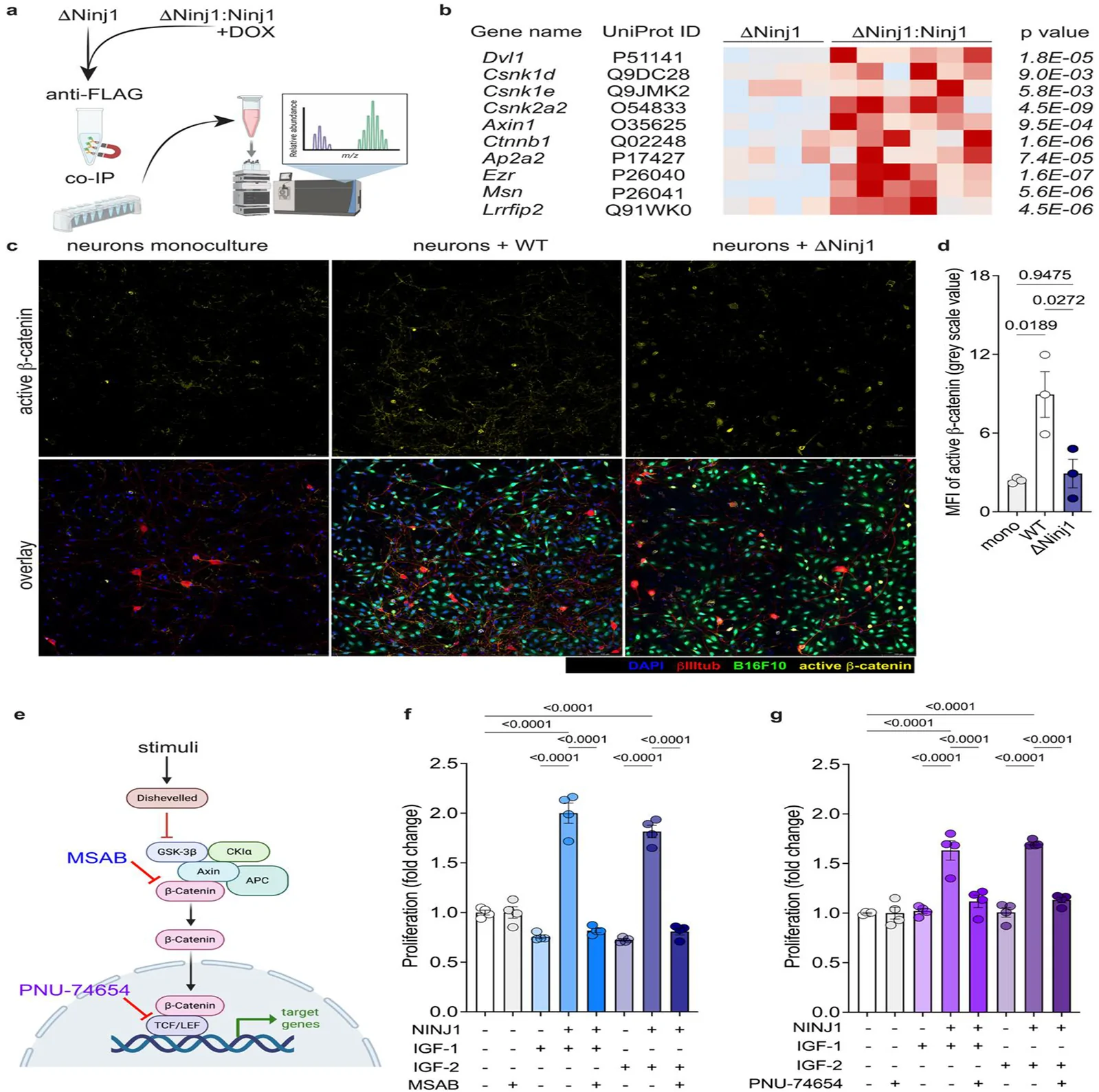
NINJ1 modulates tumor outgrowth via the β-catenin signaling pathway. **a.** Schematic representation of anti-FLAG-coated magnetic beads co-immunoprecipitation (co-IP) of B16F10^ΔNinj1^ and B16F10^ΔNinj1:Ninj1^ followed by liquid chromatography-tandem mass spectroscopy (LC-MS/MS)-based proteomics. **b.** Heatmap of molecules enriched in B16F10^ΔNinj1:Ninj1^ co-IP samples, showing gene names, UniProtID, and p-values. n = 4-6 per group. **c.** Representative images of active β-catenin staining in vagal ganglia neurons monoculture or co-cultured with B16F10^WT^ and B16F10^ΔNinj1^ cells. **d.** Quantitation of active β-catenin mean fluorescence intensity (MFI). n = 3 per group. **e.** Schematic representation of Wnt/β-catenin signaling pathway and the target MSAB and PNU-74654. MSAB binds to β-catenin and promotes its degradation, whereas PNU-74654 inhibits β-catenin nuclear translocation and binding to TCF/LEF transcription factor. **f.** Quantification of B16F10^ΔNinj1:Ninj1^ cell proliferation following MSAB, IGF-1, IGF-2, or NINJ1 (induced by DOX 18 μg/mL). n = 4 per group. **g.** quantification of B16F10^ΔNinj1:Ninj1^ proliferation following PNU-74654, IGF-1, IGF-2, or NINJ1 (induced by DOX 18 μg/mL). n = 4 per group. Data are shown as mean ± SEM. Statistical significance was determined by unpaired two-tailed Student’s t-test (b) and one-way ANOVA with Tukey’s post hoc test (d, f, and g). DOX, doxycycline.

NINJ1 was originally discovered as a pro-regenerative molecule required for nerve repair following injury. While this role has been attributed to its activity as a cell adhesion molecule, its potential role as a signaling molecule and transcriptional regulator remains largely unexplored. We next sought to investigate the signaling properties of NINJ1, identify potential molecular mechanisms, and determine whether these mechanisms operate in tumor cells and sensory neurons. To this end, we performed co-immunoprecipitation using anti-FLAG–coated magnetic beads in the cell lysates of B16F10^ΔNinj1^ and B16F10^ΔNinj1:Ninj1^ (FLAG-tagged NINJ1 rescue) (Fig. 5a). The NINJ1 co-precipitated proteins were subsequently analyzed by liquid chromatography–tandem mass spectrometry (LC–MS/MS) (Fig. 5a). Proteomic analysis revealed enrichment of key components of the Wnt/β-catenin signaling pathway among the precipitated proteins, including its major upstream signaling scaffold, Dishevelled (DVL1), and key members of the β-catenin destruction complex, including AXIN1, casein kinase 1δ/ε, and casein kinase 2 (Fig. 5b). The results suggest that NINJ1 functions as a positive regulator of β-catenin signaling by sequestering critical elements of the destruction complex responsible for directing β-catenin to proteasomal degradation. Importantly, none of the receptors for the growth factors that we tested (IGF-1 and 2) were detected co-immunoprecipitated with NINJ1, indicating that NINJ1 does not act as a co-receptor for these factors. This observation is consistent with our previous findings, which demonstrated that NINJ1 serves as a transcriptional regulator within the Wnt/β-catenin pathway and its associated target genes (Fig. 4a-b). Using scRNA-seq, we also found that lung metastasis tumors from sensory neuron-depleted animals (Nav1.8-DTA) exhibited lower Wnt/β-catenin module scores (Extended Data Fig. 8a-b), indicating convergence with signaling downstream of NINJ1. Hence, we further detected an increase in activated β-catenin protein when cancer cells were co-cultured with vagal sensory neurons, and this required the expression of NINJ1 by cancer cells (Fig. 5c-d, Expanded Data Fig. 9a-c). Additionally, we also observed that the cancer-neuron interaction mutually affected each other, with the presence of cancer cells driving neurite outgrowth of vagal sensory neurons via NINJ1 (Extended Data Fig. 9d-h). This effect is consistent with the establishment of a bidirectional, contact-dependent mechanism as previously described in Schwann cells after nerve injury, corroborating the idea that cancer cells use NINJ1 to co-opt neural repair programs. To test the involvement of β-catenin signaling in NINJ1’s effects, we used two inhibitors of the β-catenin pathway: MSAB, which binds to β-catenin and induces its degradation^60^, and PNU-74654, which blocks β-catenin translocation to the nucleus and its interaction with the T-cell factor (Tcf) ^61^ (Fig. 5e). Both strategies completely blocked the NINJ1-enabled responsiveness of cancer cells to growth factors (IGF-1- and IGF-2) (Fig. 5f-g), confirming that the effects of NINJ1 are mediated by activation of the β-catenin signaling pathway. Together, these results uncover a neural repair program exploited by metastatic cells through the expression of NINJ1 to initiate a cell-intrinsic transcriptional program that confers a stem-like identity, which in turn sensitizes the cancer cell to growth factors, ultimately enabling it to hijack the pro-regenerative local stromal cells to fuel its own expansion.

## DISCUSSION

Recent studies have begun to implicate vagal sensory and vagal brain–body circuits in cancer-associated immune regulation and systemic disease, including tumor immune evasion and the neural control of cancer-associated cachexia^62,63^. These findings establish vagal afferents as active contributors to cancer biology beyond their known physiological roles in host defense and tissue repair. Our results extend this emerging framework by identifying a distinct, contact-dependent mechanism through which metastatic cancer cells directly engage vagal sensory neurons to co-opt an intrinsic neural repair program. Unlike previously described mechanisms centered on neuropeptide signaling or systemic inflammatory reflexes, the NINJ1-dependent neuron–tumor interaction described here enables cancer cells to directly stabilize β-catenin signaling and exploit local stromal and immune-derived trophic cues to overcome the post-extravasation bottleneck. While the nervous system is increasingly recognized as an active participant in tumorigenesis, research has largely focused on the role of efferent autonomic nerves or the paracrine action of diffusible neurotransmitters. Here, we investigate a distinct modality and test the hypothesis that metastatic cells engage vagal sensory neurons through a membrane-bound molecule, hijacking their intrinsic repair programs to facilitate colonization. This line of inquiry presents a fascinating paradox, as it posits a pro-tumorigenic role for vagal sensory afferents, contrasting with the well-established protective, anti-inflammatory functions of vagal efferent pathways.

Our results demonstrate that vagal sensory neurons actively support metastatic colonization in the lung across multiple tumor types and genetic backgrounds. Depletion of Nav1.8^+^ or TRPV1^+^ neurons – whether via genetic, AAV-mediated, surgical ablation, or pharmacologic denervation – consistently reduced lung metastatic burden, underscoring a generalizable role for this neuronal population in facilitating metastasis outgrowth. These findings point to lung-innervating vagal sensory neurons as critical enablers of metastatic seeding and expansion. Mechanistically, we identify NINJ1 as a central adhesion molecule that mediates the physical tethering of metastatic cancer cells to sensory neurons. Both tumor-intrinsic and neuron-derived NINJ1 were required for metastatic outgrowth, and genetic or pharmacologic disruption of NINJ1 at either interface markedly impaired lung colonization. Beyond adhesion, NINJ1 reprograms metastatic cells to acquire a pro-regenerative, stem-like phenotype, characterized by downregulation of differentiation markers and enhanced responsiveness to stromal growth factors, particularly IGF-1 and IGF-2. This NINJ1-dependent responsiveness mimics a classic tissue repair program, co-opted by metastatic cells in the absence of injury, to exploit the pro-survival niche ^64^, reframing the hijacked program as a coordinated tissue response. The NINJ1-mediated neuron-cancer interaction appears to be the critical event that grants the cancer cell illegitimate access to a pre-existing, multi-cellular tissue repair program. This engagement allows the metastatic cell to thrive within a pro-survival and pro-proliferative niche—supported by stromal cells like macrophages and fibroblasts—that would normally be mobilized to support the regeneration of damaged tissue. These findings align with extensive evidence identifying tumor-associated macrophages (TAMs) ^65^ and cancer-associated fibroblasts (CAFs)^66^ as key architects of a pro-tumorigenic niche, in part through their secretion of growth factors. Originally described as an alveolar macrophage-derived growth factor in the lung, IGF-1 is mainly produced by pro-regenerative (M2-like) tissue macrophages and known to promote the growth and survival of cancer cells ^67-69^. Thus, NINJ1 emerges as a dual-function molecule: a neuronal adhesion scaffold and a transcriptional coadjutant of stem-like signature and growth factor sensitivity. These findings uncover a previously unappreciated neuro-interaction axis that promotes metastasis and nominate NINJ1 as a tractable target for disrupting neuron-tumor crosstalk in the metastatic niche.

This mechanism challenges the prevailing view that neuronal support for tumors is primarily mediated by soluble factors (*e.g.,* neuropeptides) acting directly in the tumor cell^70-72^ or by modulating anti-tumor immunity^73-76^. While previous studies have shown that tumors can respond to neurotrophins or neurotransmitters ^21,74,76,77^, our findings reveal a hitherto unrecognized mechanism in which neuronal contact can directly influence the tumor cell metastatic outgrowth and transcriptional state. The identification of NINJ1 as a bidirectional contact molecule in neuron-tumor interactions parallels its established role in nerve injury and regeneration, suggesting that metastatic cells may hijack neuronal repair pathways to seed and expand in a foreign microenvironment. Other studies have previously demonstrated the correlation of NINJ1 expression levels and worse prognosis in non-small cell lung cancer ^56,78^. A key question that remains is the intracellular signaling pathway that connects the cell-surface adhesion event mediated by NINJ1 to the profound transcriptional reprogramming observed in the cancer cell nucleus. The mechanisms uncovered in this study may intersect with the Wnt/β-catenin signaling pathway, which was previously implicated in NINJ1-driven tumor progression and which our transcriptomic analysis suggests is downregulated in tumor cells lacking NINJ1. Pharmacologic targeting of Wnt/β-catenin has been explored in multiple malignancies, however, no FDA-approved therapies currently exist that specifically inhibit this pathway, despite several investigational agents demonstrating preclinical efficacy ^79^. In this context, NINJ1 inhibition may operate one level upstream, preventing the initiation of the Wnt/β-catenin-driven transcriptional program by disrupting the neuron-tumor or tumor-tumor cohesion events that may trigger it. Such an approach could block both the physical scaffolding and the downstream transcriptional reprogramming that collectively promote metastatic outgrowth.

From a translational perspective, NINJ1 is an attractive therapeutic target. Both antibody-mediated neutralization and synthetic peptide blockade significantly reduced lung metastases in our models, highlighting the feasibility of disrupting neuron-tumor interaction *in vivo*. Given the role of NINJ1 in nerve repair, the safety profile of systemic inhibition will require careful evaluation, and strategies that achieve tumor-specific or neuron-tumor interface-specific targeting may offer the greatest therapeutic index. Combination approaches pairing NINJ1 blockade with immunotherapies, TAMs, or CAFs modulating agents may further enhance efficacy. Crucially, the functional outcome of NINJ1 expression (pro- vs. anti-tumorigenic) has been shown to be dependent on the p53 status of the cell. The cell lines used in this study have distinct p53 pathway statuses: B16F10 is WT p53 but p19ARF-null (functionally attenuated p53), 4T1 is p53-null, and EO771 is p53-mutant. The pro-metastatic role of NINJ1 observed across all three models may therefore be a specific feature of cancers with compromised p53 function.

In the 19^th^ century, Rudolf Virchow proposed that inflammation fuels tumorigenesis. In the 20^th^ century, Harold F. Dvorak described cancer as ‘a wound that does not heal’. In line with these ideas, our data suggests that metastatic cells do not simply mimic a wound but actively co-opt a multi-cellular tissue repair program initiated by vagal sensory neurons. By engaging NINJ1, metastatic cells effectively trick the nervous system into perceiving a state of injury, thereby gaining access to the pro-regenerative niche normally reserved for tissue restoration. This converts the neuron-cancer-stroma interface into a supportive ecosystem for tumor growth. Thus, our study reframes the view of metastasis as the pathological appropriation of homeostatic repair mechanisms and underscores the potential of targeting NINJ1 to disrupt this aberrant 'healing' process at its inception.

Although our findings suggest a critical role for vagal sensory neurons and NINJ1-mediated interaction in metastatic progression, some limitations should be acknowledged. First, we relied primarily on experimental metastasis models, in which tumor cells are introduced intravenously, bypassing several early steps of the metastatic cascade, including intravasation and escape from the primary tumor. While these models provide high reproducibility and control over metastatic timing and site, they may not fully capture the complexity of spontaneous tumor evolution and dissemination. Future studies using genetically engineered mouse models or orthotopic models that develop spontaneous metastases will be essential to validate the relevance of neuron-tumor interactions across the entire metastatic timeline. Additionally, the biases inherent to current metastasis models – such as preferential homing to the lungs and reliance on specific tumor cell lines – may limit the generalizability of our findings to other organs and cancer types. While we observed consistent results across multiple cancer models (melanoma, triple-negative, and luminal B–like breast cancers), whether NINJ1-mediated neural interactions operate similarly in non-lung tissues remains to be explored. The role of other non-neuronal cells expressing NINJ1 (e.g. Schwann cells) in metastatic outgrowth cannot be ruled out. Finally, our data support a model in which NINJ1 promotes metastatic fitness by enhancing tumor cell responsiveness to growth factors through β-catenin activation and nuclear translocation. This mechanism initially supports metastatic niche establishment through NINJ1-dependent tumor-neuron interactions and subsequently engages neuronal growth programs to drive neuronal activation, neurite outgrowth, and metastatic foci expansion. Thus, our findings identify NINJ1 as an upstream regulator of β-catenin signaling in both tumor cells and sensory neurons, establishing a targetable bidirectional signaling axis that promotes metastatic outgrowth and tumor-associated sensory innervation.

## METHODS

### Animals and Experimental Model

Mice were housed in a temperature- and humidity-controlled facility under a 12-hour light–dark cycle, with ad libitum access to food and water. Both male and female mice, aged 2 to 3 months at the onset of experiments, were used throughout the study. No sex-based differences were observed, and data from both sexes were pooled. The following strains were used: C57BL/6 (WT; JAX 000664), B6.129P2-Gt(ROSA)26Sor^tm1(DTA)Lky^/J (Rosa-DTA; JAX 009669), B6.129(Cg)-Scn10a^tm2(cre)Jwo^/TjpJ (Nav1.8-cre, JAX 036564); B6(Cg)-Tyr^c-2J^/J (albino C57BL/6, JAX 000058), BALB/cJ (Balb/c, JAX 000651). Mice were bred in-house or purchased from The Jackson Laboratory (JAX). Sample sizes were determined by power analysis using estimates from prior studies and are described in the figure legends. All procedures were approved by the Institutional Animal Care and Use Committee (IACUC) from Washington University in St. Louis.

### Sensory Neuron Depletion

Nav1.8 sensory neurons were genetically ablated by crossing B6.129P2-Gt(ROSA)26Sor^tm1(DTA)Lky^/J (Rosa-DTA; JAX 009669) with B6.129(Cg)-Scn10a^tm2(cre)Jwo/^TjpJ (Nav1.8-Cre; JAX 036564). Offspring carrying both alleles (Nav1.8-Cre^+/−^Rosa-DTA^+/−^) exhibited selective ablation of Nav1.8^+^ sensory neurons. Littermates lacking Cre recombinase were used as controls. Genotypes were confirmed by TaqMan-based PCR before inclusion in the experiments (TransnetYX, Cordova, TN). TRPV1^+^ sensory neurons were ablated using resiniferatoxin (RTX) as previously described^29,80^.

Briefly, four-week-old mice received subcutaneous injections of 100, 70, and 30 μg/kg of RTX on three consecutive days or vehicle (2% DMSO in PBS). Mice were monitored for adverse effects and allowed to recover for 4 weeks before experimentation. Vehicle-injected mice were used as controls. TRPV1^+^ neurons were also selectively ablated using an AAV2/9-TRPV1-DTA viral construct (produced by BrainVTA, Wuhan, China). Mice received 2 × 10^10^ viral units (vu) per site. Intratracheal instillation (i.t.) was performed under 3% isoflurane anesthesia, delivering 100 μL of viral suspension in Opti-MEM followed by 300 μL of air using an endotracheal intubation kit (Kent Scientific, Torrington, CT, cat. # ETI-MSE). Intra-ganglial injection (i.g.) was carried out bilaterally into the vagal (nodose/jugular) ganglia using 1 μL of virus per ganglion. Mice were anesthetized with an intraperitoneal injection of ketamine (100 mg/kg) and xylazine (10 mg/kg). Mice were allowed to recover for 4 weeks before inclusion in experiments to ensure stable neuronal depletion. Control animals received the empty vector (AAV2/9-TRPV1) via the same routes. The specificity and efficiency of TRPV1+ sensory-fiber depletion were assessed by CGRP immunohistochemistry in the vagal ganglia and thoracic dorsal root ganglia (DRG).

### Unilateral Cervical Vagotomy

Unilateral cervical vagotomy was performed under isoflurane anesthesia (3% induction, 1.5–2% maintenance). Mice were placed in a supine position, and a ~1 cm midline cervical incision was made to expose the right cervical vagus nerve. Using fine forceps under a dissecting microscope, the connective tissue was gently separated, and the nerve was carefully isolated from the carotid artery. The right vagus nerve (ipsilateral) was transected with fine microsurgical scissors, while the left vagus nerve (contralateral) was left intact to serve as an internal control. For sham-operated controls, the right cervical vagus nerve was exposed and isolated as described above, but not transected. In both groups, the incision was closed with absorbable 6-0 sutures, and mice were placed on a warming pad until fully recovered from anesthesia. Postoperative analgesia was provided using extended-release buprenorphine (1 mg/kg, subcutaneous) immediately after anesthesia recovery. Animals were monitored daily for signs of distress or impaired feeding. Mice were allowed a two-week recovery period prior to intravenous tumor cell injection for metastasis experiments.

### Cell lines and culture

The following cell lines were used in this study: B16F10-Luciferase-eGFP (Imanis Life Sciences, Rochester, MN, cat# CL068), EO771-Luciferase-mCherry2 (gift from Jason Weber), 4T1-Luciferase-eGFP (gift from Katherine N. Weilbaecher), murine immortalized bone marrow-derived macrophages (NR-9456, BEI Resources), and fibroblasts NIH3T3 (ATCC, Cat. #CRL-1658). All cells were maintained in high-glucose complete Dulbecco’s Modified Eagle Medium (DMEM) supplemented with 10% fetal bovine serum (FBS) and 1% penicillin–streptomycin, in a humidified incubator at 37°C with 5% CO_2_. All lines were routinely tested and confirmed negative for mycoplasma contamination. Cells were passaged 2–3 times per week and used at passages 3–10 for all experiments. Cell identity was confirmed by morphology and growth characteristics, and cell viability was routinely assessed by trypan blue exclusion. For *in vivo* and *in vitro* studies, cells were harvested using 0.05% trypsin-EDTA (ThermoFisher Scientific, Waltham, MA, cat# 25300062), or TrypLE^™^ Express Enzyme (ThermoFisher Scientific, Waltham, MA, cat# 12605028), washed twice in sterile 1X PBS, counted using a mirrored hemocytometer, and resuspended at the specified concentration or dose.

### Cancer Lung Metastasis Model

To induce experimental lung metastasis, mice were injected via the lateral tail vein with tumor cells resuspended in sterile 1X PBS: B16F10-Luciferase-eGFP and EO771-Luciferase-mCherry2 cells were administered at 6 × 10^5^ cells in 200 μL 1X PBS, while 4T1-Luciferase-eGFP cells were injected at 1 × 10^5^ cells in 200 μL 1X PBS. Cell suspensions were freshly prepared, and viability was confirmed before injection. Bioluminescence imaging was performed on days 7 and 14 post-injection using an i*n vivo* imaging system (IVIS^™^, PerkinElmer/Revvity, Waltham, MA) to assess metastatic burden. On day 15, mice were euthanized via intraperitoneal injection of Fatal-Plus (pentobarbital sodium, 3.90 mg/mL, 100 μL per 10 g body weight). To remove circulating immune cells, mice were perfused with 10 mL of ice-cold 1X PBS containing heparin (50 U/mL) through the left ventricle (systemic circulation), followed by 10 mL through the right ventricle (pulmonary circulation). Lungs were collected either into complete DMEM (10% FBS) for immediate dissociation and flow cytometry or fixed in 4% paraformaldehyde (PFA) for immunohistochemistry (IHC), unless otherwise stated. No significant changes in body weight were observed throughout the 15-day experimental period, indicating that the administered tumor cell doses were well tolerated. All imaging and endpoint analyses were performed with the experimenter blinded to group allocation.

### Precision-cut lung slices (PCLS)

Precision-cut lung slices (PCLS) were prepared as described^81^ with minor adaptations. Wild-type mice were euthanized with Fatal-Plus and transcardially perfused with 10 mL sterile 1X PBS via peristaltic pump (2 mL min^−1^). The trachea was left intact, cannulated with a 22-gauge catheter, and the lungs were gently inflated with 1 mL low-melting-point (LMP) agarose (2% in 1X PBS) pre-warmed to 40 °C. Under a dissecting scope, residual clots and the trachea were removed, and the lobes separated. The individual lobes were embedded in 40°C LMP agarose (8% in 1X PBS) and polymerized at 4°C for 4 min. Agarose blocks were mounted on a vibrating microtome (VT1000S; Leica) and sectioned at 250 μm in ice-cold Krebs buffer. The slices were collected into 5 mL Hibernate at 4 °C before being transferred to 5 mL adult Neurobasal media (Gibco, ThermoFisher, Cat. # 10888022) supplemented with 2% B27 for 35 min at 37 °C (5% CO_2_) to recover. For co-culture, PCLS were transferred to 35-mm dishes pre-seeded with a monolayer of 1×10^5^ cancer cells (B16F10-eGFP or EO771-mCherry2). Slices were placed directly onto the cells and incubated without medium for 3 min at 37 °C, 100% humidity to promote adherence, then overlaid with a thin collagen solution (v/v: 10.4% 10× MEM, 1.9% penicillin–streptomycin, 4.2% sodium bicarbonate, 83.5% collagen [PureCol^®^, Advanced Biomatrix, Cat. # 5005]) and polymerized for 10 min at 37 °C, 5% CO_2_. Subsequently, 0.8 mL of 2% B27 Neurobasal A medium was added to establish an air–liquid interface, and media were changed daily. Three random fields per slice were imaged at prespecified times (or at 72 hours) under identical settings, and the tumor area was quantified in ImageJ/Fiji by threshold-based segmentation by a blinded analyst. Data are presented as mean ± standard error of the mean (SEM) of the number of cells per region of interest (ROI) or the fluorescent protein area per μm^2^ as a surrogate of tumor outgrowth.

### Bioluminescence imaging

*In vivo* bioluminescence imaging was performed on the 7th and 14th days after tumor i.v. injection on an IVIS 50 (IVIS^™^, PerkinElmer/Revvity, Waltham, MA; Living Image 4.3.1, 1 min (7 days) or 10 sec (14 days) exposures, 8 bin, FOV12cm, f/stop1, open filter). Mice were injected intraperitoneally with D-luciferin (150mg/kg in 1X PBS; Gold Biotechnology, St. Louis, MO) and imaged 10 minutes later using isoflurane anesthesia (2% vaporized in O_2_). Total radiance (photons/sec/cm^2^/sr) was measured from fixed ROIs over the mouse thorax using Living Image 2.6. The results are expressed as mean ± standard error of the mean (SEM) of Photon Flux (photons/second).

### Public cancer dataset analysis

To assess NINJ1 expression across human cancers, publicly available RNA-seq datasets were analyzed using GEPIA3, an online platform integrating data from TCGA and GTEx ^82^. The boxplot module was used to compare *NINJ1* mRNA expression between tumor and normal tissues across the indicated cancer types using default parameters. Plots were exported directly from the GEPIA3 web interface. GEPIA3 was accessed on March 11^th,^ 2026. The analyzed cancer types are listed in the corresponding figure legend.

### NINJ1 Neutralization and Blocking

To neutralize NINJ1 *in vivo*, mice were treated with a mouse anti-NINJ1 monoclonal antibody (BD Biosciences, Cat. #610777, RRID: AB_398098) ^83-85^ or an isotype-matched IgG control (BioLegend, Cat. #400202, RRID: AB_2927399). Treatments were administered at a dose of 2.5 μg per animal in 100 μL 1X PBS via intraperitoneal (i.p.) injection, beginning immediately after tumor cell injection via the tail vein, and repeated every 3 days for 15 days. For peptide-mediated NINJ1 blockade, mice received either a NINJ1 blocking peptide^85,86^ (NINJ1_26–37_, sequence: PPRWGLRNRPIN, purity >98%) or a scrambled control peptide (PWPPRRNRNGLI, purity >97%), both of which were custom synthesized (GenScript Biotec, Piscataway, NJ). Peptides were administered at 1 mg/kg or 10 mg/kg in 100 μL of sterile 1X PBS (i.p.), using the same dosing schedule as the neutralizing antibody treatment. All treatments were prepared and coded independently, and the experimenter was blinded to group assignment and treatment allocation.

### Orthotopic Subcutaneous Melanoma Model

B16F10-eGFP cells were prepared as described above (see Cell lines and culture) and resuspended in sterile 1X PBS at 2 × 10^5^ cells per 50 μL. Cells were injected subcutaneously into the popliteal fossa. Tumor progression was monitored by in vivo bioluminescence imaging (IVIS^™^) every 7 days. Mice were treated with either anti-NINJ1 or an isotype-matched IgG control (2.5 μg in 100 μL, i.p.) every 3 days. Fifteen days after tumor-cell injection, tumors were excised, weighed, and measured with calipers. Tumor volume was calculated using the standard formula: $\left(\text{length}\;\times \;{\text{width}}^{2}\right)∕2$. Data are presented as mean ± standard error of the mean (SEM).

### *Ninj1* Knockdown in Vagal Sensory Neurons

To specifically downregulate neuronal *Ninj1* expression, we used a recombinant AAV construct (rAAV-CMV-DIO-(EGFP-U6)-shRNA(*Ninj1*)-WPRE-hGH polyA) encoding a short hairpin RNA targeting 5′-GCTGCTCATCTTCCTGGTCAA-3′. The shRNA and a scrambled shRNA control were designed and produced by BrainVTA (Wuhan, China). Viral particles (2 x 10^10^ vg per animal) were delivered intratracheally to Nav1.8-Cre^+/−^ mice, enabling Cre-dependent expression of EGFP and sh*Ninj1* selectively in Nav1.8^+^ sensory neurons. Metastasis was induced 4 weeks after AAV administration. The specificity and efficiency of *Ninj1* knockdown were assessed by immunohistochemistry in the vagal ganglia and thoracic dorsal root ganglia (DRG).

### CRISPR/Cas9-Mediated Generation of m*Ninj1* Knockout Cell Lines

m*Ninj1* knockout (KO) cell lines were generated by the Genome Engineering & Stem Cell Center (GESC@MGI) at Washington University in St. Louis. A synthetic guide RNA (gRNA) targeting the *Ninj1* gene (sequence: 5′-GCCGGTTCCGCAAACCCCAGNGG) was complexed with recombinant Cas9 protein. The Cas9-gRNA ribonucleoprotein (RNP) complex was transfected into B16F10, 4T1, or EO771 cells. Transfected cells were single-cell sorted into 96-well plates, and individual clones were expanded. Clonal knockout lines were validated by next-generation sequencing (NGS) to detect out-of-frame indels, confirming *Ninj1* disruption (**Supplementary Table 2**). Additionally, short tandem repeat (STR) profiling was performed to authenticate the identity of each cell line (**Supplementary Table 3**).

### Lung Single-Cell Suspension Preparation

Whole lungs were collected in ice-cold complete DMEM (10% fetal bovine serum [FBS], and 1% Penicillin/Streptomycin [P/S]). Tissues were transferred to the lid of a 15 mL conical tube, and excess medium was removed using sterile gauze. Lungs were minced into small fragments (2–5 mm) and transferred to the original 15 mL tube containing 2 mL of pre-warmed digestion buffer composed of DMEM supplemented with 2% FBS, 1% P/S, 1 mg/mL collagenase VIII (Sigma-Aldrich, Cat. #C2139), and 0.5 mg/mL DNase I (Roche, Cat. #04536282001)^87^. Samples were incubated for 30 minutes at 37 °C in a water bath with occasional agitation. Following enzymatic digestion, tissues were mechanically dissociated by pipetting up and down using a 3 mL syringe fitted with an 18G needle. The resulting cell suspension was filtered through a 70 μm cell strainer, and enzymatic activity was neutralized by adding 2 mL of complete medium (DMEM + 10% FBS). An additional 2 mL of FACS buffer was then added, and the samples were centrifuged (400 × g, 5 minutes, 4 °C). Pellets were resuspended in 1 mL of FACS buffer and kept on ice until further processing.

### Flow cytometry

The lung single-cell samples were transferred (1/10) into a U-bottom 96-well plate. Cell viability was assessed using Zombie NIR (1:500 in 1X PBS, 15 min, room temperature, BioLegend Cat. # 423106). Samples were centrifuged (400 × g, 5 minutes, 4 °C) and resuspended in anti-CD16/32 antibody (1:100, BioLegend, Cat. # 101302, RRID:AB_312801) diluted in FACS buffer to block Fc receptor binding. Samples were stained with BV-750 rat anti-CD45 (BioLegend, Cat. #103157, RRID:AB_2734155), BUV563 rat anti-CD11b (BD, Cat. #741242, RRID:AB_2870793), BV421 armenian hamster anti-CD11c BioLegend, Cat. #117343, RRID:AB_2563099), Pacific Blue rat anti-Ly6C (BioLegend, Cat. #128014, RRID:AB_1732079), BV605 rat anti-F4/80 (BioLegend, Cat. # 123133, RRID:AB_2562305), BV650 rat anti-CD3 (BioLegend, Cat. # 100229, RRID:AB_11204249), BV711 rat anti-CD8 (BioLegend, Cat. # 100759, RRID:AB_2563510), BV785 rat anti-CD19 (BioLegend, Cat. # 115543, RRID:AB_11218994), PE rat anti-CD4 (BioLegend, Cat. # 130310, RRID:AB_2075573), PE-Cy5 rat anti-Ly6G (BioLegend, Cat. # 127672, RRID:AB_2904289), PE-Cy7 rat anti-CD206 (BioLegend, Cat. # 141720, RRID:AB_2562248), and Alexa Fluor 647 mouse anti-NK1.1 (BioLegend, Cat. # 108720, RRID:AB_2132713), or PerCP/Cyanine5.5 anti-DYKDDDDK Tag (BioLegend, Cat. #637326, RRID:AB_2750065) for 30 minutes on ice. Flow cytometry was performed using a Cytek^®^ Aurora 5L spectral flow cytometer (Cytek Biosciences, Fremont, CA), and the number of tumor cells in the lung parenchyma was analyzed with FlowJo (version 10, BD Biosciences). The gate strategy is illustrated in the support information (**Supplementary Data Figure 1**). Cell doublets were excluded by SSC-A/SSC-H (SC1) and FSC-A/FSC-H (SC-2), and only live cells (Zombie NIR negative) were analyzed. Parental tumor cell lines were used to define tumor cell gating, and mixed cultures were used for compensation controls, including eGFP-expressing (B16F10 and 4T1) and mCherry2-expressing (EO771) cells. Results are presented as mean ± standard error of the mean (SEM) of the absolute number of cells per lung.

### Single-cell RNA sequencing

Lung single-cell suspensions from tumor-bearing control and Na_v_1.8-DTA (pool of 10 mice/group) were incubated with CD45 MicroBeads (Miltenyi Biotec, Cat. #130-052-301, RRID:AB_2877061) as previously described^29^. The flow-through containing CD45^−^ cells were collected and passed through a second MS column to increase depletion efficiency. All columns were washed twice with ice-cold FACS buffer, and the combined eluates were pooled as the CD45^−^ fraction. Columns were subsequently removed from the magnetic stand, and retained CD45^+^ cells were eluted and collected into a separate tube. Both CD45^+^ and CD45^−^ fractions were centrifuged at 400 × g for 5 minutes at 4 °C and resuspended in 2 mL of 1X PBS containing 0.04% bovine serum albumin (BSA). Cell concentrations were adjusted to 1,000 cells/μL, and viability was assessed using trypan blue exclusion. Single-cell RNA sequencing was performed at the Genome Technology Access Center (GTAC@MGI), McDonnell Genome Institute, Washington University in St. Louis. Single cells were captured in emulsion droplets using the 10x Genomics Chromium Controller, followed by barcoding and cDNA synthesis. Library preparation was carried out according to the Chromium Single Cell 3' Reagent Kits v3.1 User Guide using dual indexing, and bar-coded cDNA was sequenced by NovaSeq high-throughput (1 billion reads per library, ~ 50 K reads/cell).

### Single-cell Data Analysis

Raw sequencing data were processed using the Cell Ranger pipeline (10x Genomics) for alignment, barcode assignment, and UMI counting. The resulting gene-barcode matrices were imported into R (2025.05.1+513) and analyzed using the Seurat package (v 5.1.0). Cells were filtered to remove low-quality or dying cells, retaining cells with >500 detected genes, <200,000 total unique molecular identifiers (UMIs), and <10% mitochondrial gene content. Genes expressed in fewer than 3 cells were excluded. Data were log-normalized using the *NormalizeData* function, and the top 2,000 variable features were identified with *FindVariableFeatures* using the "vst" method. Principal component analysis (PCA) was performed using RunP*CA*, and significant PCs were selected based on *ElbowPlot*. Cells were clustered using the Louvain algorithm implemented in *FindNeighbors* and *FindClusters*, with resolution parameters ranging from 0.4. For visualization, dimensionality reduction was performed using Uniform Manifold Approximation and Projection (UMAP) via *RunUMAP*. Cluster-specific marker genes were identified using *FindAllMarkers* with the Wilcoxon rank-sum test, requiring a minimum log2 fold change of 0.25 and detection in at least 25% of cells in the cluster. Significant markers were filtered based on adjusted p-value < 0.05. Gene expression visualization was performed using Seurat plotting functions (*e.g., FeaturePlot*, *DotPlot*, and *DoHeatmap)* or using the Nebulosa package^88^ (*e.g.,* plotting function *plot_density*).

Wnt/β-catenin module scores were calculated in tumor cells using *AddModuleScore* in Seurat from the gene set *Axin2*, *Lef1*, *Myc*, *Ccnd1*, and *Tcf7*. Scores were compared between control and Nav1.8-DTA cells within each tumor cluster (Tumor I, Tumor II, and Tumor III) using a two-sided Wilcoxon rank-sum test. P values were corrected for multiple comparisons across clusters using the Benjamini–Hochberg method. Center lines indicate medians; boxes indicate the interquartile range; violin plots show the distribution of single-cell scores.

### Computational Prediction of Neuron-Tumor Interactions

To predict molecular interactions between metastatic tumor cells and sensory neurons, we integrated our scRNA-seq data with a publicly available dataset of murine vagal neurons (GEO accession: GSE124312). The raw expression matrix and metadata for the vagal neurons were downloaded and processed into a Seurat object. This neuron object was then merged with our scRNA-seq object containing the tumor cell clusters. Gene expression data was normalized using Seurat’s NormalizeData function. Average expression profiles for the "Tumor" and "Neuron" populations were calculated and exported. These profiles were used as input for an interaction prediction workflow in Python. The OmniPath database^89^ was queried via its web service to retrieve a comprehensive set of known protein-protein interactions. The analysis was filtered to retain high-confidence interactions relevant to intercellular communication, including ligand-receptor pairs and adhesion molecules as defined by OmniPath's intercell annotations. Potential interactions were further filtered to include only pairs where the proteins were expressed in tumor cells and in neurons above an average normalized expression threshold of 1.0. The final network of predicted interactions was visualized as a bipartite graph using the networkx library in Python. The expression distribution of *Ninj1* across the merged single-cell populations was visualized using the VlnPlot function in Seurat.

### Bulk RNA sequencing

B16F10 wild-type (B16F10^WT^) and NINJ1 knockout cells (B16F10^ΔNinj1^) (3 samples per group, 1 × 10^6^ cells per sample) were collected as pellets and submitted to the Genome Technology Access Center (GTAC@MGI), McDonnell Genome Institute, Washington University in St. Louis. RNA was extracted, and poly(A) mRNA enrichment was performed. RNA quality was assessed prior to library preparation, and all samples had a RIN of 10, followed by library preparation and high-throughput sequencing. Aligned sequencing files (BAM format) were generated using the GTAC@MGI standard pipeline and uploaded to Partek^®^ Flow^®^ software (Illumina Inc., San Diego, CA) for downstream analysis. Gene-level counts were normalized, and differential gene expression analysis was performed using the DESeq2 algorithm. Genes with an adjusted p-value (FDR) < 0.05 were considered significantly differentially expressed.

### Gene Ontology (GO) Enrichment Analysis

Gene Ontology (GO) enrichment analysis was performed using the Database for Annotation, Visualization and Integrated Discovery (DAVID) v6.8 (NIH, https://david.ncifcrf.gov/). A list of downregulated genes identified from the comparison between B16F10 and NINJ1 knockout (KO) cells was uploaded to DAVID, using Mus musculus as the background genome. Enrichment analysis was carried out using the Gene Ontology Biological Process (GO BP) category. Default settings were used for statistical thresholds and multiple testing correction. Significantly enriched GO terms were defined based on a Benjamini-adjusted p-value (FDR) < 0.05.

### Quantitative Reverse Transcription PCR (qRT-PCR)

Total RNA was extracted using TRIzol^™^ Reagent (Thermo Fisher Scientific) according to the manufacturer’s instructions and previously described ^90^. Briefly, RNA concentration and purity were assessed using a NanoDrop^™^ spectrophotometer (Thermo Fisher Scientific). For cDNA synthesis, 1,000 ng of total RNA was reverse transcribed using the GoScript^™^ Reverse Transcriptase Kit (Promega, Cat. #A5001) with a combination of oligo(dT) and random primers, following the manufacturer’s protocol. Quantitative PCR was performed using the GoTaq^®^ qPCR and RT-qPCR System (Promega, Cat. #A6001). Each reaction contained 50 ng of cDNA template and was run in triplicate under standard cycling conditions. Primer sequences used for target gene amplification are listed in **Supplementary Table 4**. Gene expression levels were calculated using the comparative Ct (ΔΔCt) method, with normalization to appropriate housekeeping genes.

### Immunohistochemistry

Lungs, vagal ganglia, and dorsal root ganglia (DRG) were harvested and drop-fixed in 4% paraformaldehyde (PFA) in 1X PBS for 24 hours at 4 °C. Fixed tissues were washed in 1X PBS and either stored in 1X PBS with 0.05% sodium azide for later processing or cryoprotected by incubation in 30% sucrose in 1X PBS for 48 hours at 4 °C. Samples were then snap-frozen in OCT compound and sectioned using a cryostat: 50 μm for lungs and 16 μm for vagal ganglia and DRG. Lung sections were stored in 1X PBS with 0.05% sodium azide, while ganglia sections were mounted directly onto glass slides. Lung sections underwent antigen retrieval in 10 mM sodium citrate buffer (pH 6.0) for 30 minutes at room temperature, followed by washing in 1X PBS and incubation with 0.1 mM glycine in 1X PBS to quench autofluorescence. Vagal and DRG sections were rehydrated in 1X PBS before staining. All tissues were blocked in 5% BSA with 0.3% Triton X-100 in 1X PBS for 1 hour at room temperature—on slides for ganglia and in 48-well plates for lung sections in suspension. To reduce background, samples were then incubated with a mouse-on-mouse IgG blocking reagent (Thermo Fisher, Cat. #R37621) for 1 hour at room temperature. Primary antibodies were incubated overnight at 4 °C for ganglia and DRG, and for 72 hours at 4 °C for lung sections. The following primary antibodies were used: CGRP (Sigma-Aldrich, Cat. #C8198, RRID: AB_259091, 1:500), NINJ1 (BD Biosciences, Cat. #610777, RRID: AB_398098, 1:200), βIII-Tubulin (Abcam, Clone 2G10, Cat. #AB78078, RRID: AB_2256751; and Clone EPR19591, Cat. #AB215037, 1:500), βIII-Tubulin (Millipore, Clone TU-20, Cat. # MAB1637, RRID: AB_2210524; 1:100), active β-catenin (Cell Signaling Technologies, Cell Signaling Technology Cat # 8814, Clone D13A1, Cat. # 8814, RRID: AB_11127203; 1:400). After primary incubation, tissues were washed three times in 1X PBS with 0.3% Triton X-100 and incubated with the appropriate species-specific secondary antibodies: Goat anti–Guinea Pig IgG Alexa Fluor 647 (Thermo Fisher, Cat. #A21450, RRID: AB_2535867, 1:500), Goat anti–Rabbit IgG TRITC (Thermo Fisher, Cat. #T2769, RRID: AB_2556777, 1:500), and Goat anti–Mouse IgG Alexa Fluor 647 (Thermo Fisher, Cat. #A21235, RRID: AB_2535804, 1:500). Following final washes, sections were mounted with Fluoromount-G containing DAPI (SouthernBiotech). Imaging was performed on a widefield fluorescence microscope (Olympus) or a confocal microscope (MICA, Leica). The same acquisition settings were applied across all experimental groups to ensure comparability. Colocalization analyses were performed using Imaris Software (Oxford Instruments, Abingdon, United Kingdom; v.11), considering I) eGFP (whole tumor cell) and DAPI (tumor cell and neuronal nuclei). Fluorescence intensity was analyzed by LAS X Software (Leica Application Suite X; v.5.3.1). The experimenter was blinded for image acquisition and data analysis.

### Cell Proliferation Assay

B16F10, EO771, and 4T1 wild-type (WT) and NINJ1 knockout (ΔNinj1) cells were transduced with IncuCyte^®^ NucLight Red Lentivirus 2.0 (Sartorius, Göttingen, Germany, Cat. #BA-04889) according to the manufacturer’s instructions. Cells expressing red fluorescent nuclei (mCherry2) were selected by fluorescence-activated cell sorting (FACS) using a BD FACSymphony^™^ S6 Cell Sorter (Becton Dickinson, Franklin Lakes, NJ) and subsequently cultured in phenol red-free complete DMEM. Cell proliferation was monitored using the IncuCyte^®^ S3 Live-Cell Analysis System (Sartorius, Göttingen, Germany). A total of 20,000 cells were seeded per well in 96-well plates and maintained at 37 °C in a humidified atmosphere with 5% CO_2_. Cells were treated with vehicle (culture media), 1 ng/mL of IGF-1 (B16F10 and EO771), 100 ng/mL of IGF-1 (4T1), 10 ng/mL of IGF-2 (B16F10, EO771, and 4T1), TGF-b 30-300 pg/mL (B16F10) or 300 pg/mL (4T1), or FGF-1 1-10 ng/mL (B16F10). Phase-contrast and red fluorescence images were captured every hour (B16F10 and EO771) or every two hours (4T1) for 36 hours to assess proliferation. For each well, the data represent the average of three fields analyzed per time point. Four to eight biological replicates (*n* = 4–8) were included per experimental group. Proliferation was quantified as a fold change, calculated by dividing the number of red nuclei at each time point by the baseline measurement (time 0). Data are presented as fold change compared to vehicle-treated wild-type cells as mean ± standard error of the mean (SEM).

### Doxycycline-Inducible NINJ1 Expression in Cancer Cells

To generate a doxycycline (DOX)-inducible NINJ1 expression system, we used the pRetroX-TRE3G_NINJ1 plasmid (Addgene, Plasmid #201181), previously described ^91^. The plasmid encodes murine NINJ1 tagged with a C-terminal FLAG epitope under the control of a tetracycline response element. The plasmid was expanded in LB broth containing ampicillin (100 μg/mL) and purified using a QIAprep Spin Miniprep Kit (Qiagen), according to the manufacturer’s instructions. For retroviral production, HEK293T-Ampho cells were transfected with the plasmid using polyethylenimine (PEI). After 48 hours, the culture supernatant containing viral particles was collected, filtered (0.45 μm), and used to transduce NINJ1-knockout B16F10 cells (B16F10^ΔNinj1^) in the presence of 8 μg/mL polybrene. Following transduction, cells were selected with puromycin (2 μg/mL) for 3–5 days to establish stable lines. Expression of NINJ1-FLAG upon DOX induction (18 μg/mL, 24 h) was confirmed by flow cytometry using an anti-FLAG antibody (BioLegend, Cat. #637326, RRID:AB_2750065).

### Co-immunoprecipitation and LC–MS/MS

B16F10^ΔNinj1^ and B16F10^ΔNinj1:Ninj1^ (FLAG-tagged NINJ1 rescue) cells were lysed in ice-cold lysis buffer (50 mM Tris-HCl pH 7.5, 150 mM NaCl, 1% NP-40, supplemented with protease and phosphatase inhibitors). Lysates were clarified by centrifugation (10,000 × g, 15 min, 4 °C). Protein concentration was determined by BCA assay (Thermo Fisher Scientific, Cat. # A55865), and equal amounts of protein were used for each immunoprecipitation. FLAG-tagged proteins were immunoprecipitated using anti-DYKDDDDK magnetic beads (Pierce^™^, Thermo Fisher Scientific, Cat. #A36797) by incubating lysates overnight at 4 °C with gentle rotation. Beads were washed extensively (3 times) with lysis buffer and 1X PBS (5 times with tube change) to remove non-specifically bound proteins and detergent. Bound proteins were subjected to on-bead digestion. Briefly, proteins were reduced, alkylated, and digested with sequencing-grade trypsin overnight at 37 °C. Peptides were collected and desalted using C18 spin columns before analysis. Peptides were analyzed by liquid chromatography–tandem mass spectrometry (LC–MS/MS) using a data-independent acquisition (DIA) method. Raw data were processed using Spectronaut software (Biognosys) and searched against the UniProt *Mus musculus* protein database. Label-free quantification (LFQ) was performed using default settings in Spectronaut. A total of four biological replicates for B16F10^ΔNinj1^ and six biological replicates for B16F10^ΔNinj1:Ninj1^ were included in the analysis. Proteins detected in the FLAG-tagged rescue condition were compared to ΔNinj1 controls to identify NINJ1-associated proteins.

### Macrophage Polarization and Co-cultures Assay

Murine immortalized bone marrow-derived macrophages (NR-9456, BEI Resources) were polarized toward M2-like (recombinant IL-4 [20 ng/mL]) for 24 hours. Following polarization, macrophages were harvested, washed, and co-cultured with B16F10^WT^, B16F10^ΔNinj1^, or B16F10^ΔNinj1:Ninj1^ cells. Co-cultures were established by seeding equal numbers (50,000 cells each) of macrophages and tumor cells in 24-well plates and incubating for 24 hours under standard conditions (37 °C, 5% CO_2_). In another set of experiments, tumor cells were co-cultured with fibroblasts (NIH-3T3) in the same conditions. At the end of the co-culture period, cell proliferation was assessed by flow cytometry. Singlets were identified by sequential gating on FSC-H/FSC-A and SSC-H/SSC-A, followed by exclusion of dead cells using Zombie NIR. Tumor and non-tumor populations were then distinguished based on eGFP intensity, with tumor cells defined as eGFP-positive and macrophages or fibroblasts as eGFP-negative. Data are presented as the mean ± SEM of fold change in the number of viable eGFP+ tumor cells relative to monoculture controls.

### Vagal ganglion neuron–tumor co-culture

Primary vagal sensory neurons were isolated from WT mice nodose ganglia and cultured as previously described ^29,30,90,92^. Briefly, vagal ganglia (nodose/jugular) were dissected under sterile conditions, enzymatically digested using collagenase A (1 mg/mL; Roche, Cat. #10103586001) and dispase II (3 mg/mL; Roche, Cat. #04942078001) and mechanically dissociated (up-and-down pipetting) to obtain a single-cell suspension. Neurons were plated on mouse recombinant laminin (ThermoFisher Scientific, Cat. #23017015)-coated round glass cover slips and maintained in Neurobasal A medium (ThermoFisher Scientific, Cat. #21103049) supplemented with B27 (Gibco, Cat. #A3353501), nerve growth factor (NGF, 50 ng/mL; Sigma-Aldrich, Cat. # N6009-4X25UG), and arabinosylcytosine (ARA-C, 10 μM/mL; Sigma-Aldrich, Cat. #C1768) to restrain non-neuronal cell proliferation. Cultures were allowed to recover for 24 hours before being washed and co-cultured with B16F10 cells.

For co-culture experiments, B16F10^WT^ or B16F10^ΔNinj1^ tumor cells were added directly to neuronal cultures at a 1:1 ratio (10,000 tumor cells per well in 24-well plates) and maintained under standard conditions (37 °C, 5% CO_2_) in complete DMEM high glucose media supplemented with fetal bovine serum (10%) and penicillin/streptomycin (1%). Samples were fixed after 24 or 48 hours of co-culture with 4.2% formaldehyde BD Cytofix/Cytoperm (BD Biosciences, Cat. # BDB555028) for 20 min at 37 °C. Samples were washed with 1X PBS and submitted to immunohistochemistry for active β-catenin and βIII-tubulin.

### Neurite Outgrowth

The branch length, number of trees, junctions and endpoints of neurons were analyzed using fully automated macro in ImageJ (Fiji x64). Briefly, the immunohistochemistry images acquired using a confocal microscope (MICA, Leica; 10X magnification) were processed to 8-bit grayscale images for thresholding (min=5 and max=90), followed by skeletonization and 2D skeleton analyses.

### Statistical analysis

For statistical comparison of two groups, either a two-tailed Student’s t-test or a Mann–Whitney U test was used, depending on data distribution. Normality was assessed using the D’Agostino–Pearson omnibus test and the Shapiro–Wilk test. If the data followed a normal distribution, Student’s t-test was applied; otherwise, the nonparametric Mann–Whitney test was used. For comparisons involving more than two groups with one independent variable, one-way ANOVA was performed, followed by Dunnett’s or Tukey’s multiple comparison tests as specified in the figure legends. If normality assumptions were not met, the Kruskal–Wallis test with Dunn’s multiple comparisons test was used. For experiments involving two independent variables, two-way ANOVA was conducted, followed by Tukey’s, Bonferroni’s, or Šidák’s multiple comparison tests, as indicated in the respective figure legends. All data are presented as mean ± standard error of the mean (SEM) and are representative of two independent experiments. Statistical analyses were performed using GraphPad Prism 9.4.0 (GraphPad Software), except for single-cell RNA sequencing analyses, which were conducted in R using RStudio (version 2025.05.1+513). Detailed information on sample sizes (n), number of independent experiments, and animal sex is provided in the corresponding figure legends.

## Extended Data

## Supplementary Material

This is a list of supplementary files associated with this preprint. Click to download.

- Zaninellietal.SupplementaryInformation.docx
- SupplementaryDataVideo1.mp4

Supplementary Information is available for this paper. For further information and requests, please contact the corresponding author, Felipe Almeida de Pinho-Ribeiro (dfelipe@wustl.edu).
